# Low-pressure versus standard-pressure pneumoperitoneum in laparoscopic cholecystectomy: a systematic review and meta-analysis of randomized controlled trials

**DOI:** 10.1007/s00464-022-09201-1

**Published:** 2022-04-18

**Authors:** Monica Ortenzi, Giulia Montori, Alberto Sartori, Andrea Balla, Emanuele Botteri, Giacomo Piatto, Gaetano Gallo, Silvia Vigna, Mario Guerrieri, Sophie Williams, Mauro Podda, Ferdinando Agresta

**Affiliations:** 1 Clinica di Chirurgia Generale e d’Urgenza, Università Politecnica delle Marche, Ancona, Italy; 2 Emergency Department, Leopoldo Mandic Hospital, Merate, LC Italy; 3 U. O. Chirurgia Generale e d’urgenza, Ospedale San Valentino - Montebelluna, Montebelluna, Treviso Italy; 4 UOC of General and Minimally Invasive Surgery, Hospital “San Paolo”, Largo Donatori del Sangue 1, 00053 Civitavecchia, Rome, Italy; 5 ASST Soedali Civili Brescia, Montichiari, PO Italy; 6 Department of Medical and Surgical Sciences, University of Catanzaro, Catanzaro, Italy; 7 U. O. Chirurgia Generale Ospedale Civile, Cittadella, Padua, Italy; 8 Department of Colorectal Surgery, King’s College Hospital, London, UK; 9 Department of Surgical Science, University of Cagliari, Cagliari, Italy; 10 Department of General Surgery, AULSS2 del Veneto, Vittorio Veneto, TV Italy

**Keywords:** Pneumoperitoneum, Laparoscopic cholecystectomy, Low-pressure pneumoperitoneum, Standard-pressure pneumoperitoneum, Clinical outcomes, Systematic review, Meta-analysis

## Abstract

**Introduction:**

It has been previously demonstrated that the rise of intra-abdominal pressures and prolonged exposure to such pressures can produce changes in the cardiovascular and pulmonary dynamic which, though potentially well tolerated in the majority of healthy patients with adequate cardiopulmonary reserve, may be less well tolerated when cardiopulmonary reserve is poor.

Nevertheless, theoretically lowering intra-abdominal pressure could reduce the impact of pneumoperitoneum on the blood circulation of intra-abdominal organs as well as cardiopulmonary function. However, the evidence remains weak, and as such, the debate remains unresolved. The aim of this systematic review and meta-analysis was to demonstrate the current knowledge around the effect of pneumoperitoneum at different pressures levels during laparoscopic cholecystectomy.

**Materials and methods:**

This systematic review and meta-analysis were reported according to the recommendations of the 2020 updated Preferred Reporting Items for Systematic reviews and Meta-analyses (PRISMA) guidelines, and the Cochrane handbook for systematic reviews of interventions.

**Results:**

This systematic review and meta-analysis included 44 randomized controlled trials that compared different pressures of pneumoperitoneum in the setting of elective laparoscopic cholecystectomy. Length of hospital, conversion rate, and complications rate were not significantly different, whereas statistically significant differences were observed in post-operative pain and analgesic consumption. According to the GRADE criteria, overall quality of evidence was high for intra-operative bile spillage (critical outcome), overall complications (critical outcome), shoulder pain (critical outcome), and overall post-operative pain (critical outcome). Overall quality of evidence was moderate for conversion to open surgery (critical outcome), post-operative pain at 1 day (critical outcome), post-operative pain at 3 days (important outcome), and bleeding (critical outcome). Overall quality of evidence was low for operative time (important outcome), length of hospital stay (important outcome), post-operative pain at 12 h (critical outcome), and was very low for post-operative pain at 1 h (critical outcome), post-operative pain at 4 h (critical outcome), post-operative pain at 8 h (critical outcome), and post-operative pain at 2 days (critical outcome).

**Conclusions:**

This review allowed us to draw conclusive results from the use of low-pressure pneumoperitoneum with an adequate quality of evidence.

**Supplementary Information:**

The online version contains supplementary material available at 10.1007/s00464-022-09201-1.

Minimally invasive surgery (MIS) has enabled a dramatic change in the management of most gastrointestinal surgical pathology, through improving post-operative pain and reducing recovery time [1, 2]. The establishment and maintenance of a stable pneumoperitoneum is an integral part of MIS [1, 2]; it is essential to create sufficient operative space in order to safely manipulate the instruments. Traditionally, standard-pressure pneumoperitoneum for laparoscopic cholecystectomy is considered to be about 15 mmHg [3].

It has been previously demonstrated that the rise of intra-abdominal pressures and prolonged exposure to such pressures can produce changes in the cardiovascular and pulmonary dynamic which, though potentially well tolerated in the majority of healthy patients with adequate cardiopulmonary reserve, may be less well tolerated when cardiopulmonary reserve is poor. In such cases, laparoscopic procedures may be avoided due to the potential adverse outcomes resulting from significant changes in the cardiovascular and pulmonary dynamic. There are several studies demonstrating changes in metabolic, humoral, and neurological systems following high-pressure pneumoperitoneum. [4–8]

Nevertheless, theoretically lowering intra-abdominal pressure could reduce the impact of pneumoperitoneum on the blood circulation of intra-abdominal organs as well as cardiopulmonary function. Furthermore, some patients experience unpleasant post-surgical symptoms such as shoulder pain, seemingly specific to laparoscopic surgery [1, 9]. Approximately one-third of the patients undergoing a laparoscopic procedure develop this complaint postoperatively [1, 10]. The origin of shoulder pain is only partly understood, but it is commonly assumed that the cause is overstretching of the diaphragmatic muscle fibres owing to a high rate of insufflation [11]. Other causes, including peritoneal stretching and diaphragmatic irritation, have also been considered. [12]

When considering such theories, potential solutions must also be postulated. Reducing insufflation pressure to improve post-operative outcomes seems a logical hypothesis.

Nevertheless, the evidence remains weak, and as such, the debate remains unresolved. In clinical practice, many surgeons continue to use high pneumoperitoneum pressures mainly due to personal preference and belief rather than due to scientific evidence.

The aim of this systematic review and meta-analysis was to demonstrate the current knowledge around the effect of pneumoperitoneum at different pressures levels during laparoscopic cholecystectomy.

## Materials and methods

This systematic review and meta-analysis were conducted according to the recommendations of the 2020 updated Preferred Reporting Items for Systematic reviews and Meta-analyses (PRISMA) guidelines [13], and the Cochrane handbook for systematic reviews of interventions [14].

## Criteria for considering studies for the review

### Types of studies

**Fig. 1:**
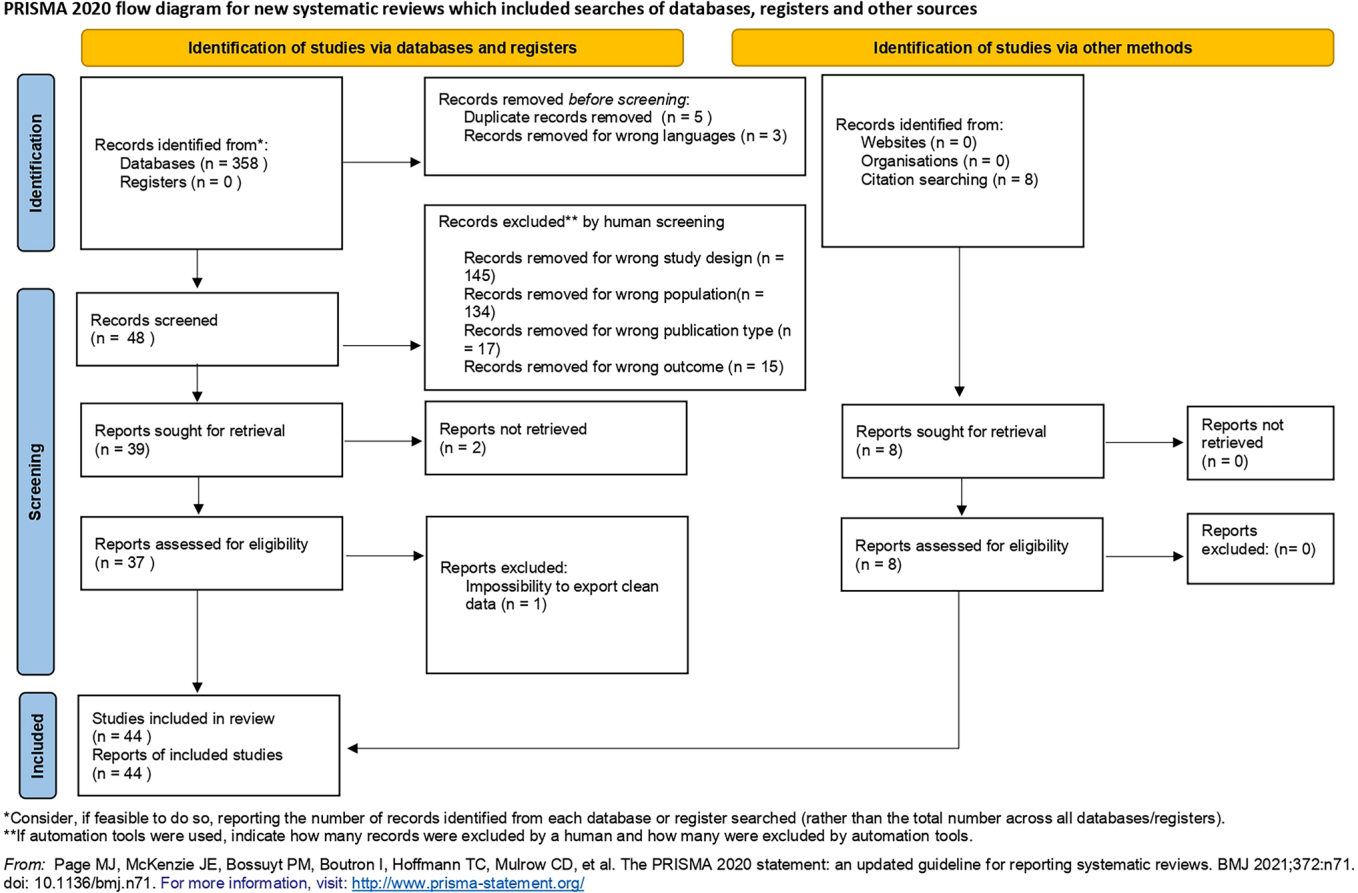
PRISMA flow diagram

This systematic review and meta-analysis included 44 randomized controlled trials that compared different pressures of pneumoperitoneum in the setting of elective laparoscopic cholecystectomy. Most of the studies have compared two study groups (low- vs standard- or high-pressure pneumoperitoneum) [1, 4, 15–48], whereas 7 studies included three or more study groups, as reported in Table 1 [49–56] (Fig. 1).

**Table 1:** Characteristics of RCTs included in the systematic review

Author (year) [Refs]	Country	Duration of study	*N* of randomized Pts (pts include in the study)	IAP in study arms (mmHg)		*N* of Pts for arm	
				LPG	SPG/HPG	LPG	SPG/HPG
Chock (2006) [15]	China	Jen 2004–Dec 2004	40	7	12	20	20
Ekici (2009) [16]	Turkey	Oct 2006–Nov 2007	52	7	15	20	32
Ibraehim (2006) [17]	Saudi Arabia	NR	20	6–8	12–14	10	10
Joshipura (2009) [18]	India	Oct 2006–Oct 2007	46 (26)	8	12	14	12
Koc (2005) [19]	Turkey	Jen 2002–Oct 2002	53 (50)	10	15	25	25
Perrakis (2003) [20]	Greece	May 2001–Oct 2001	40	8	15	20	20
Wallace (1997) [21]	UK	Sep 1994–May 1997	40	7.5	15	20	20
Zaman (2015) [22]	India	Jul 2014–Mar 2015	50	7–8	12–14	25	25
Ali (2016) [1]	Pakistan	Jen 2013–Aug 2013	160	≤ 10	> 10	80	80
Barczynski (2002) [27]	Poland	NR	20	7	10	10	10
Barczynski (2003) [28]	Poland	May 2000–Dec 2001	148	7	12	74	74
Bhattacharjee (2017) [29]	USA	Nov 2014–Sep 2015	80	9–10	14	40	40
Karagulle (2009) [30]	Turkey	NR	30	8	12	15	15
Kanwer (2009) [31]	India	Jul 2006–Jun 2007	60	7–10	14	30	30
Morino (1998) [32]	Italy	Sep 1995–Mar 1996	32	10	14	22	22
Hasukič (2005) [23]	Bosnia	May 2001–Dec 2001	50	7	14	25	25
Donmez (2016) [24]	Turkey	Jul 2015–Jan 2016	50	10	14	25	25
Filho (2021) [25]	Brazil	Jan 2018–Jan 2020	64	6–8	10–12	33	31
Dexter (1999) [26]	UK	NR	20	7	15	10	10
Gupta (2013) [36]	India	Jan 2011–Dec 2011	101	8	14	50	51
Goel (2019) [37]	India	Sept 2017–Dec 2018	60	7–10	12–14	30	30
Gin (2021) [53]	Australia	Feb 2019–Oct 2019	100	8	12	51	49
Ko-iam (2016) [38]	Thailand	Jan 2012–Mar 2014	120	7	14	60	60
Mohammadzade (2016) [39]	Iran	2012	60	7–10	12–14	30	30
Nasajiyan (2014) [40]	Iran	Dec 2012–Sept 2013	50	7–9	14–15	25	25
Singla (2014) [41]	India	NR	100	7–8	12–14	50	50
Shoar (2015) [42]	Iran	NR	50	8	12	25	25
Torres (2009) [43]	Poland	Jan 2006–Mar2006	40	6–8	12–14	20	20
Yasir (2012) [44]	India	Nov 2009–Oct 2010	100	8	14	50	50
Vijayaraghavan (2012) [45]	India	NR	43	8	12	22	21
Sarli (2000) [47]	Italy	Jan 1998–Jul 1998	90	9	13	46	44
Sandhu (2008) [48]	Thailand	Jan 2003–Nov 2003	140	7	14	70	70
Neogi (2019) [4]	India	NR	80	7	14	32	48
Basgul (2004) [33]	Turkey	Mar 2001–Ape 2001	22	10	14–15	11	11
Polat (2003) [35]	Turkey	NR	24	10	15	12	12
Sefr (2003) [46]	Czech Republic	Jen 1999–Jul 1999	30	10	15	15	15
Eryılmaz (2012) [34]	Turkey	NR	43	10	14	20	23

*Pts* patients, *IAP* intra-abdominal pressure, *vs* versus, *N* number, *LPG* low-pressure group, *HPG* high- pressure group, *SPG* standard-pressure group

### Types of participants

All the papers but one [51] included patients undergoing elective laparoscopic cholecystectomy, and one study also included patients undergoing emergency laparoscopic cholecystectomy [51]. Surgical indications were different: symptomatic gallstones, acalculous cholecystitis, gallbladder polyps, or any other condition. We applied no restriction based on the type of anaesthesia or patient positioning used, reporting that the same type of anaesthesia was used in both groups.

### Types of interventions

Thirty-seven trials compared low-pressure (≤ 10 mmHg) versus standard- or high-pressure (> 10 mmHg) pneumoperitoneum [15–48]. Seven trials compared three or more pressure groups as reported in Table 1 (Barrio (2017) [49], Celik (2010) [50], Kandil et al. (2010) [51], Esmat (2006) [52], Gin (2021) [53], Umar (2013) [54], Topal (2011) [55], and Celik (2004) [56]). Pneumoperitoneum pressure < 6 mmHg or > 15 mmHg was not reported by any of the included trials. The definitions of “low”, “standard”, and “high” pressure were established by the review's authors using web-based discussion and brainstorming, as no universal definitions are available in the literature.

### Types of outcome measures

According to the PICO criteria, we included general and clinical primary outcomes into the analysis: post-operative abdominal pain (assessed with the Visual Analogue Scale VAS) and shoulder pain, analgesic use, surgical morbidity, length of hospital stay (LOS), conversion rate (laparoscopic to open, or from low to standard/high pneumoperitoneum pressure), operative time, quality of life, and surgeon satisfaction. Secondary outcomes, defined as “functional”, were respiratory function, cardiac function, liver function, and inflammatory response.

## Search methods for the identification of studies to be included in the review

A computerized search was performed in MEDLINE (via PubMed), EMBASE, and the Cochrane Central Register of Controlled Trials databases for articles published from 1992 to 2021.

The literature search was carried out according to the primary search strategy: “Laparoscopy OR Laparoscopic surgery AND Low-pressure pneumoperitoneum OR Low pressure pneumoperitoneum OR Ultra-low pneumoperitoneum pressure OR Low-pressure laparoscopy AND Standard pressure pneumoperitoneum OR Normal pressure pneumoperitoneum”.

The studies identified by the primary search strategy were subsequently selected based on title, abstract, and full-text review by two independent reviewers (M.P. and G.M.) in Rayyan web app for systematic reviews (https://www.rayyan.ai/). Articles published in languages other than English, non-randomized studies, and animal and preclinical studies were excluded. Reference lists of relevant studies were searched manually, and the “related articles” function in PubMed was used.

## Risk of bias assessment in the included studies

The risk of bias in the included randomized controlled trials was independently assessed by two authors (G.M and M.O.) using the Risk of bias assessment (RoB-2) tool without masking the trial names. The methodological quality of the RCTs was assessed based on sequence generation, allocation concealment, blinding of participants, personnel, and outcome assessors, incomplete outcome data, selective outcome reporting, and other sources of bias. Trials that were classified as low risk of bias in sequence generation, allocation concealment, blinding, incomplete data, and selective outcome reporting were judged at low bias risk.

## Measures of treatment effect

We planned to use intention-to-treat analysis if such analysis was available from the included studies. All statistical analyses were performed using Reviewer Manager software (Reviewer Manager—RevMan—version 5.4.1, Sept. 2020, The Nordic Cochrane Centre, Cochrane Collaboration, www.training.cochrane.org). The relative risk (RR) with 95% confidence interval (95% CI) was calculated for dichotomous variables, and the standardized mean difference (SMD), with 95% CI for continuous variables. Whenever continuous data were reported as medians and range, the method of Hozo et al. to estimate respective means and standard deviations was applied [57]. The point estimate of the RR value was considered statistically significant at *P* level < 0.05 if the 95% CI did not cross the value 1. The point estimate of the SMD value was considered statistically significant at *P* level < 0.05 if the 95% CI did not cross the value 0. Statistical heterogeneity of the results across studies was assessed using the Higgins' *I*^2^ statistic and Chi-Square test. A *P* value of Chi-Square test < 0.10 with an *I*^2^ value > 30% were considered as indicative of substantial heterogeneity. Moreover, both clinical (variability in the baseline characteristics of the participants, interventions, and outcomes studied) and methodological (variability in the study design and risk of bias) heterogeneities were considered to inform the decision to use the fixed- or random-effects model. Fixed-effects model (Mantel–Haenszel) was used if significant heterogeneity was absent, whereas a random-effects model was implemented for meta-analysis if significant heterogeneity was found, according to the method of DerSimonian and Laird [58]. We constructed a funnel plot to explore the risk of publication bias in the presence of at least 10 trials for the outcome. Asymmetry in the funnel plot of trial size against treatment effect was used to assess this bias.

## Results of the systematic review

### Results of the meta-analysis

**Fig. 2:**
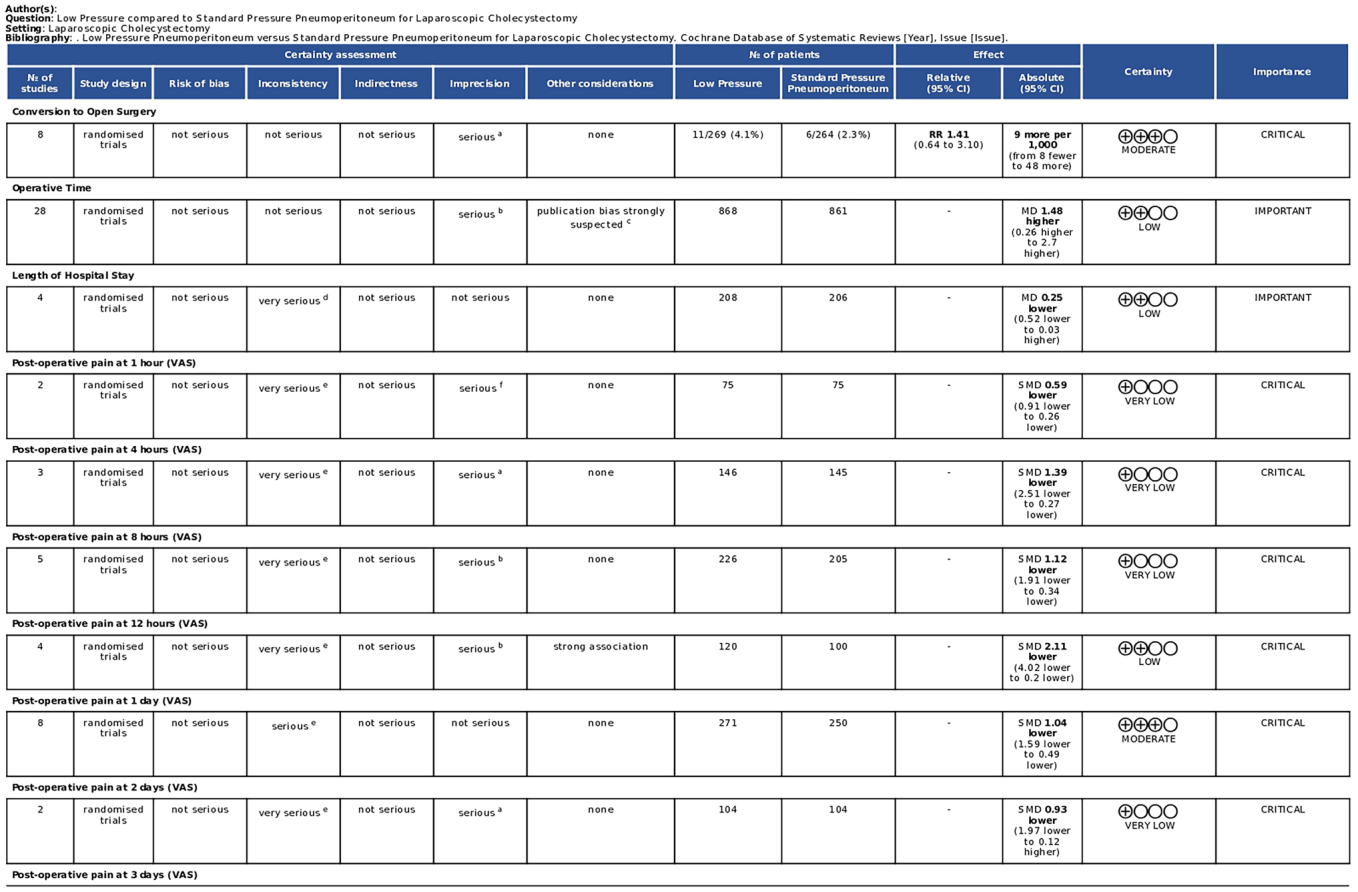
Overall study quality according to grade criteria

The results of the pooled analyses ae summarized in the summary of findings table prepared using GRADEPro (https://gradepro.org/cite/gradepro.org.) [59] Fig. 2.

### Length of hospital stay

**Fig. 3:**
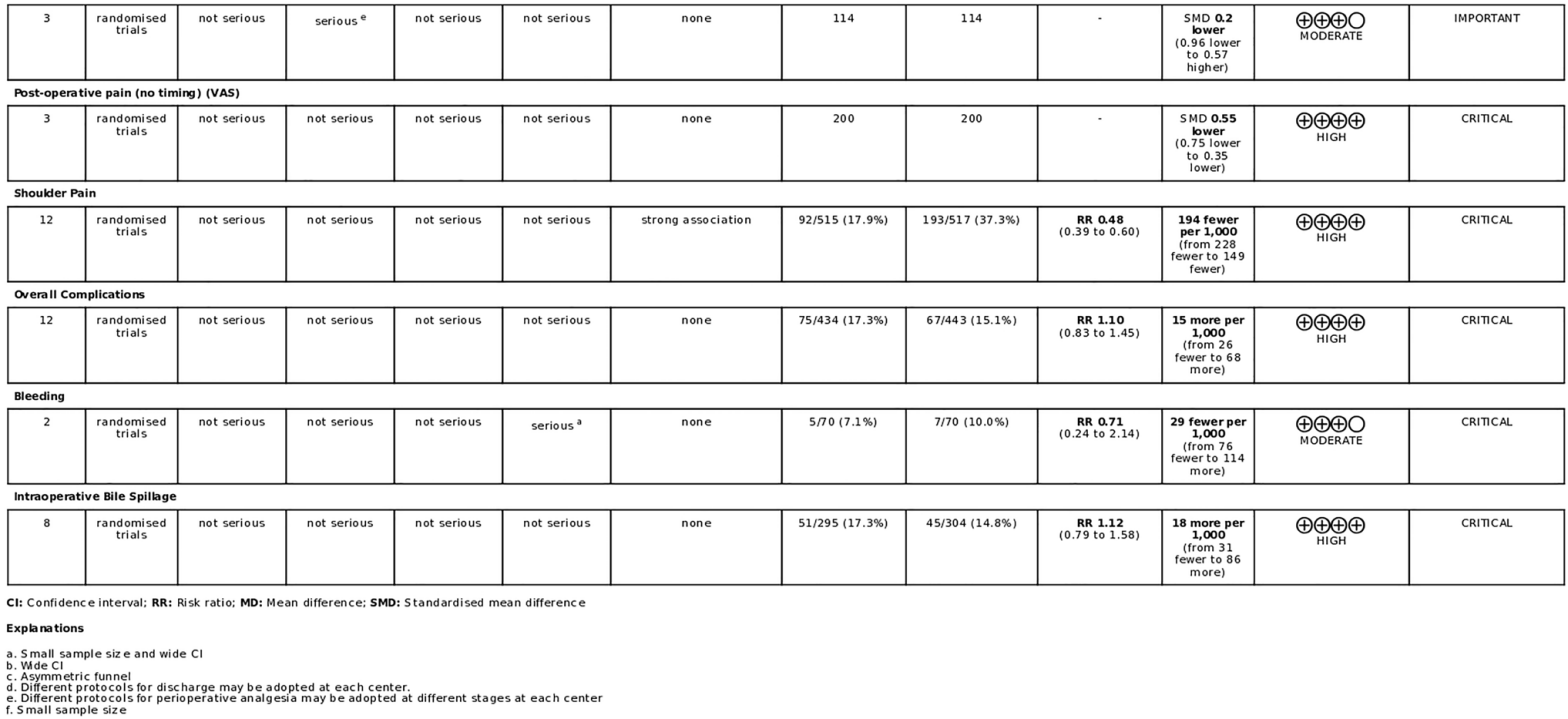
length of hospital stay

Length of hospital stay was reported in four studies (Barczynskyi 2003[28]; Joshipura 2009[18]; Sandhu 2008[48]; Yasir 2012[44]). LOS was slightly shorter in the low-pressure group than in the standard-pressure group (4 studies, 414 patients; MD − 0.25, 95% CI − 0.52 to 0.03; *I*^2^ = 91%, Random-effects), however, this difference was not statistically significant (Fig. 3).

### Conversion to open surgery

**Fig. 4:**
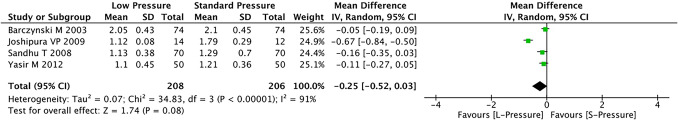
conversion to open surgery

Conversion to open surgery was reported in seven studies (Dexter 1999; Goel 2019; Kanwer 2009; Karagulle 2009; Ko-lam 2016; Sandhu 2008, Vijayaraghavan 2012). No statistically significant difference was found between the two groups (8 studies, 533 patients; RR 1.41, 95% CI 0.64 to 3.10; *I*^2^ = 10%, Fixed effects) (Fig. 4).

### Operative time

Operative time was reported in 28 studies (Ali 2016; Barczynski 2003; Basgul 2004; Bhattacharjee 2017; Chock 2006; Dexter 1999; Donmez 2016; Ekici 2009; Eryilmaz 2012; Goel 2019; Gupta 2013; Hasukic 2005; Ibraehim 2006; Joshipura 2009; Kanwer 2009; Karagulle 2009; Koc 2005; Ko-lam 2016; Nasajiyan 2014; Perrakis 2003; Polat 2003; Sandhu 2008; Sefr 2003; Shoar 2015; Singla 2014; Vijayaraghavan 2012; Wallace 1997; Yasir 2012). Mean operative time was significantly shorter in the standard-pressure group than in the low-pressure group (28 studies, 1729 patients; MD 1.48, 95% CI 0.26 to 2.70; *I*^2^ = 42%, Random effects) (Online Fig. 5)

### Post-operative pain at 1 h (VAS)

Post-operative pain at 1 h was reported in 2 studies (Singla 2014; Zaman 2015). Patients in the low-pressure group reported lower VAS compared with patients in the standard-pressure group (2 studies, 150 patients; SMD − 0.59, 95% CI − 0.91 to − 0.26; *I*^2^ = 0%, Random effects), with a statistically significant difference (Online Fig. 6)

### Post-operative pain at 4 h (VAS)

Post-operative pain at 4 h was reported in 3 studies (Barczynski 2003; Singla 2014; Vijayaraghavan 2012). Patients in the low-pressure group reported lower VAS compared with patients in the standard-pressure group (3 studies, 291 patients; SMD − 1.39, 95% CI − 2.51 to − 0.27; *I*^2^ = 94%, Random effects), with a statistically significant difference (Online Fig. 7).

### Post-operative pain at 8 h (VAS)

Post-operative pain at 8 h was reported in 5 studies (Ali 2016; Barczynski 2003; Kanwer 2009; Vijayaraghavan 2012; Wallace 1997). Patients in the low-pressure group reported lower VAS compared with patients in the standard-pressure group (5 studies, 431 patients; SMD − 1.12, 95% CI − 1.91 to − 0.34; *I*^2^ = 91%, Random effects), with a statistically significant difference (Online Fig. 8).

### Post-operative pain at 12 h (VAS)

Post-operative pain at 12 h was reported in four studies (Goel 2019; Ibraehim 2006; Kanwer 2009; Singla 2014). Patients in the low-pressure group reported lower VAS compared with patients in the standard-pressure group (4 studies, 220 patients; SMD − 2.11, 95% CI − 4.02 to − 0.20; *I*^2^ = 93%, Random-effects), with a statistically significant difference (Online Fig. 9).

### Post-operative pain at 1 day (VAS)

Post-operative pain at 1 day was reported in eight studies (Barczynski 2003; Chock 2006; Goel 2019; Kanwer 2009; Koc 2005; Singla 2014; Vijayaraghavan 2012; Wallace 1997). Patients in the low-pressure group reported lower VAS compared with patients in the standard-pressure group (8 studies, 521 patients; SMD − 1.04, 95% CI − 1.59 to − 0.49; *I*^2^ = 87%, Random effects), with a statistically significant difference (Online Fig. 10).

### Post-operative pain at 2 days (VAS)

Post-operative pain at 2 days was reported in two studies (Barczynski 2003; Goel 2019). Patients in the low-pressure group reported slightly lower VAS compared with patients in the standard-pressure group (2 studies, 208 patients; SMD − 0.93, 95% CI − 1.97 to 0.12; *I*^2^ = 91%, Random effects), without a statistically significant difference (Online Fig. 11).

### Post-operative pain at 3 days (VAS)

Post-operative pain at 3 days was reported in three studies (Barczynski 2003; Chock 2006; Wallace 1997). No statistically significant difference was found between the two groups (3 studies, 228 patients; SMD − 0.20, 95% CI − 0.96 to 0.57; *I*^2^ = 84%, Random effects) (Online Fig. 12).

### Post-operative pain (no time-frame) (VAS)

Post-operative pain (no time-frame) was reported in three studies (Ali 2016; Sandhu 2008; Singla 2014). Patients in the low-pressure group reported lower VAS compared with patients in the standard-pressure group (3 studies, 400 patients; SMD − 0.55, 95% CI − 0.75 to − 0.35; *I*^2^ = 0%, Random effects), with a statistically significant difference (Online Fig. 13).

### Post-operative shoulder pain

Shoulder pain was reported in 12 studies (Ali 2016; Barczynski 2003; Bhattacharjee 2017; Chock 2006; Ibraehim 2006; Ko-lam 2016; Nasajiyan 2014; Perrakis 2003; Sandhu 2008; Sarli 2000; Yasir 2012; Zaman 2015). Patients in the low-pressure group reported significantly lower rates of post-operative shoulder pain compared with patients in the standard-pressure group (12 studies, 1032 patients; RR 0.48, 95% CI 0.39 to 0.60; *I*^2^ = 0%, Fixed effects) (Online Fig. 14).

### Analgesic consumption at 1 day

Analgesic consumption at 1 day was reported in 5 studies (Ali 2016; Barczynski 2003; Chock 2006; Perrakis 2003; Vijayaraghavan 2012). Patients in the low-pressure group reported significantly lower rates of post-operative analgesic consumption compared with patients in the standard-pressure group (5 studies, 431 patients; RR − 1.09, 95% CI − 1.92 to − 0.26; *I*^2^ = 93%, Fixed effects) (Online Fig. 15).

### Analgesic consumption at 3 days

Analgesic consumption at 1 day was reported in 3 studies (Barczynski 2003; Chock 2006; Perrakis 2003). Patients in the low-pressure group reported significantly lower rates of post-operative analgesic consumption compared with patients in the standard-pressure group (3 studies, 228 patients; RR 0.41, 95% CI − 1.44 to 2.25; *I*^2^ = 97%, Fixed effects) (Online Fig. 16).

### Analgesic consumption (no time-frame)

Post-operative pain (no time-frame) was reported in four studies (Sandhu 2008; Vijayaraghavan 2012; Wallace 1997; Yasir 2012). Patients in the low-pressure group reported lower analgesic consumption compared with patients in the standard-pressure group (3 studies, 323 patients; SMD − 1.20, 95% CI − 2.28 to − 0.11; *I*^2^ = 94%, Fixed effects), with a statistically significant difference (Online Fig. 17).

### Overall complications

Overall complications were reported in 12 studies (Dexter 1999; Sarli 2000; Perrakis 2003; Barczynski 2003; Joshipura 2009; Vijayaraghavan 2012; Singla 2014; Donmez 2016; Ko-lam 2016; Goel 2019; Neogi 2019; Gin 2021). The difference in the incidence of post-operative complications between the two groups was not statistically significant (12 studies, 877 patients; RR 1.10, 95% CI 0.83 to 1.45; *I*^2^ = 0%, Fixed effects) (Online Fig. 18).

### Bleeding

The occurrence of bleeding was reported in two studies (Perrakis 2003; Singla 2014). The difference in the incidence of bleeding was equivalent in the two groups (2 studies, 140 patients; RR 0.71, 95% CI 0.24 to 2.14; *I*^2^ = 0%, Fixed effects) (Online Fig. 19).

### Intra-operative bile spillage

Intra-operative bile spillage was reported in eight studies (Sarli 2000; Perrakis 2003; Joshipura 2009; Vijayaraghavan 2012; Singla 2014; Ko-lam 2016; Neogi 2019; Gin 2021). The difference in the incidence of intra-operative bile spillage was equivalent in the two groups (8 studies, 599 patients; RR 1.12, 95% CI 0.79 to 1.58; *I*^2^ = 0%, Fixed effects) (Online Fig. 20).

### Quality of Evidence assessment (GRADE)

According to the GRADE criteria, overall quality of evidence was high for intra-operative bile spillage (critical outcome), overall complications (critical outcome), shoulder pain (critical outcome), and overall post-operative pain (critical outcome). (Fig. 2) Overall quality of evidence was moderate for conversion to open surgery (critical outcome), post-operative pain at 1 day (critical outcome), post-operative pain at 3 days (important outcome), and bleeding (critical outcome). Overall quality of evidence was low for operative time (important outcome), length of hospital stay (important outcome), post-operative pain at 12 h (critical outcome), and was very low for post-operative pain at 1 h (critical outcome), post-operative pain at 4 h (critical outcome), post-operative pain at 8 h (critical outcome), and post-operative pain at 2 days (critical outcome) (Figs. 3, 4).

Most of the articles included came from Turkey (10) and India (10), followed by Iran (3), Poland (3), Italy (2), Thailand (2), UK (2), Egypt (2), Spain (1), China (1), Saudi Arabia (1), Greece (1), Pakistan (1), USA (1), Bosnia (1), Brazil (1), Australia (1), and Czech Republic (1). Thirty-seven articles (Chock 2006; Ekici 2009; Ibraehim 2006; Joshipura 2009; Koc 2005; Perrakis 2003; Wallace 1997; Zaman 2015; Ali 2016; Barczynski 2002; Barczynski 2003; Bhattacharjee 2017; Karagulle 2009; Kanwer 2009; Morino 1998; Hasukič 2005; Donmez 2016; Filho 2021; Dexter 1999; Gupta 2013; Goel 2019; Gin 2021; Ko-lam 2016; Mohammadzade 2016; Nasajivan 2014; Singla 2014; Shoar 2015; Torres 2009; Yasir 2012; Vijayaraghavan 2012; Sarli 2000; Sandhu 2008; Neogi 2019; Basgul 2004; Polat 2003; Eryılmaz 2012) out of 44 analyse results retrieved from two groups of patients, whereas the other studies use three groups of patients (Barrio 2017; Umar 2013; Esmat 2006; Kandil 2010; Celik 2010; Celik 2004; Topal 2011) (Tables 1, 2, 3, and 4).

**Table 2:** Characteristics of RCTs with more than two comparative groups included in the systematic review

Author (year) [refs]	Country	Duration of study	*N* of randomized Pts (pts include in the study)	*N* of randomized Pts (pts include in the study)	IAP in study arms (mmHg)	*N* of Pts for arm
Barrio (2017) [49]	Spain	Feb 2014 – Jan 2015	90	LP + moderate- NMB (8 mmHg)	8	30
				LP + deep- NMB (8 mmHg)	8	30
				Standard (12 mmHg)	12	30
Umar (2013) [54]	India	NR	NR	Group 1 (8–10 mmHg)	8–10	NR
				Group 2 (11–13 mmHg)	11–13	NR
				Group 3 (≥ 14 mmHg)	> 14	NR
Esmat (2006) [52]	Egypt	NR	109	High (14 mmHg)	14	34
				Low (10 mmHg)	10	37
				Low + saline (10 mmHg)	10	38
Kandil (2010) [51]	Egypt	Oct 2008-Jen 2010	100 (84)	Low (8 mmHg)	8	25
				Median (10 mmHg)	10	25
				Standard (12 mmHg)	12	25
				High (14 mmHg)	14	25
Celik (2010) [50]	Turkey	Mar 2006 – Dec 2006	64 (60)	Low (8 mmHg)	8	20
				Standard (12 mmHg)	12	20
				High (14 mmHg)	14	20
Celik (2004) [56]	Turkey	NR	100	I (8 mmHg)	8	20
				II (10 mmHg)	10	20
				III (12 mmHg)	12	20
				IV (14 mmHg)	14	20
				V (16 mmHg)	16	20
Topal (2011) [55]	Turkey	NR	60	1 (10 mmHg)	10	20
				2 (13 mmHg)	13	20
				3 (16 mmHg)	16	20

*Pts* patients, *IAP* intra-abdominal pressure, *vs* versus, *N* number, *NMB* neuromuscular blockade, *LPG* low-pressure group, *HPG* high-pressure group, *SPG* standard-pressure group, *PTC* post-tetanic count

**Table 3:** Patients characteristics of the included RCTs

Author (year) [refs]	Follow-up duration (days)	Trend^a^	Mean age (in years) ± SD		Male (%)		Mean BMI (in kg/m^2^) ± SD		ASA I *N*(%)	
			LP	S/HP	LP	S/HP	LP	S/HP	LP	S/HP
Chock (2006) [15]	30	Reverse	47.6 ± 10.0	47.2 ± 11.0	8	8	NR	NR	20	20
Ekici (2009) [16]	NR	Pts position was mainly supine; however, the head-up tilt position was used in six patients (three patients in each group)	52.2 ± 10.05	49.3 ± 12.64	2	6	28.5 ± 4.76	28.4 ± 5.13	20	32
Ibraehim (2006) [17]	NR	10-15^b^ reverse	49.9 ± 10.524	47.2 ± 6.66	3 (30)	3 (30)	26.89 ± 2.1	26.985 ± 1.9	6 (60)	1 (10)
Joshipura (2009) [18]	11 months	20^b^ reverse and 15^b^ right-side elevated position with a bag below right posterior lower chest wall	57	58	9	6	27.5 ± 1.04	26 ± 1.44	NR	
Koc (2005) [19]	1	NR	46.3 ± 15.5	47.9 ± 15.2	3	6	NR		NR (ASA I-III)	
Perrakis (2003) [20]	8–10	Reverse and left tilt in all pts	57.25 ± 13.27 Median 58.50 (range 33–79)	54.75 ± 14.14 Median 55 (range 30–79)	7	3	Median 26.39 (range 21.23–34.29)	Median 25.31 (range 19.84–43.57)	12	13
Wallace (1997) [21]	6	15^b^ reverse in 50% of pts in each group	58.5 ± 3.45 Median 59 (range 52–64)	56.5 ± 4.04 Median 56 (range 50–64)	6	4	Median 26.4 (range 24.8–28.4)	Median 25.9 (range 23.1–29.5)	18 (ASA I + II)	17 (ASA I + II)
Zaman (2015) [22]	NR	NR	NR		NR		NR		25 (ASA I + II)	
Ali (2016) [1]	1	NR	40.74 ± 12.32	41.10 ± 11.96	7 (8.8)	13 (16.2)	63.15 ± 10.98	59.61 ± 12.97	NR	
Barczynski (2002) [27]	NR	Horizontal position	45 ± 12	47 ± 14	4	5	25.48 ± 1.68	26.12 ± 2.02	NR	
Barczynski (2003) [28]	7–3 weeks	15-20^b^ Reverse (The moderate reversed Trend position was employed in 36.48% LPLC and 21.62% SPLC patients (*p* < 0.05))	48.15 ± 12.06	47.82 ± 12.58	9	10	27.52 ± 3.23	27.10 ± 3.29	52	47
Bhattacharjee (2017) [29]	Nov 2014 – Sep 2015	NR	35.32 ± 11.18	37.92 ± 9.27	NR		25.197 ± 2.6	24.66 ± 2.82	NR	
Karagulle (2009) [30]	NR	Turned to left in a 10-15^b^ reverse	47.9 ± 11.6	48.7 ± 11.9	3(20)	2(13.3)	29.1 ± 4.9	29.3 ± 5.2	NR	
Kanwer (2009) [31]	NR	NR	NR		12°		NR		NR	
Morino (1998) [32]	3	NR	NR		NR		NR		NR	
Hasukič (2005) [23]	28	NR	41.88 ± 10.82	43.15 ± 12.25	2(8)	3(12)	NR		NR	
Donmez (2016) [24]	2	Reverse and left tilt in all pts	47 ± 15	52 ± 13	5(20)	6(24)	28.1 ± 4.1	27.8 ± 4.5	12	14
Filho (2021) [25]	1	NR	49.6 ± 13.2	44.4 ± 13.5	7 (22.6)	7 (21.2)	27.9 ± 3.3	27.6 ± 4.2	NR	
Dexter (1999) [26]	2	NR	46.75 ± 15.29 Median 48 (range 19–72)	52.5 ± 12.68 Median 56 (range 27–71)	3(15)	4(20)	Median 25.4 (range 18.1–32.2)	Median 27 (range 20.1–30.9)	NR	
Gupta (2013) [36]	7	Reverse	43.46 ± 11.40	44.67 ± 14.23	10 (20)	11 (21.56)	NR		NR	
Goel (2019) [37]	2	NR	36.2 ± 2.5	35.5 ± 3	NR		NR		NR	
Gin (2021) [26]	1	NR	47.6 ± 17.1	48.7 ± 14.6	13 (25)	9 (18)	Median 30.2 (IQR 25.6, 34.9)	Median 29.4 (IQR 26.7, 34.6)	7 (14)	16 (33)
Ko-iam (2016) [38]	NR	NR	51.0 ± 13.3	52.8 ± 12.1	11 (18.3)	18 (30)	24.6 ± 4.1	24.6 ± 4.1	60 (ASA I + II)	60 (ASA I + II)
Mohammadzade (2016) [39]	NR	NR	39 ± 13.3	36.4 ± 15.8	8 (26.7)	1 (3.3)	NR		NR	
Nasajiyan (2014) [40]	NR	NR	45.1 ± 12.3	42.5 ± 16.4	0	0	NR		50 (ASA I-II)	
Singla (2014) [41]	1	NR	50.60 ± 13.95	53.76 ± 13.80	12	20	60.16 ± 9.71	59.32 ± 9.96	NR	
Shoar (2015) [42]	NR	NR	45.12 ± 13.1	40.48 ± 14.4	5 (20)	8 (32)	25.08 ± 4.90	24.88 ± 4.30	NR	
Torres (2009) [43]	2	NR	NR		NR		NR		NR	
Yasir (2012) [44]	1	NR	NR		NR		NR		NR	
Vijayaraghavan (2012) [45]	1	NR	44.5 ± 31.5–51.5	40 ± 31.5–49.5	8	9	24.35 ± 21.7–26.6	24.6 ± 22–28.65	14	14
Sarli (2000) [47]	NR	NR	49.3 (NR)	47.7 (NR)	13 (28.2)	11 (25)	NR		NR	
Sandhu (2008) [48]	NR	NR	54 ± 12.93 (NS)	55.23 ± 13.2 (NS)	9 (12.8) *p* = 0.051	18 (25.71) *p* = 0.051	NR		NR	
Neogi (2019) [4]	7	Reverse position of 30^b^ and left lateral tilt of 35^b^	39.68 ± 10.45	37.79 ± 16.11	2 (6.2)	3 (6.2)	26.52 ± 3.21	26.11 ± 3.32	20 (62.5)	32 (66.6)
Basgul (2004) [33]	1	15-20^b^	48.64 ± 6.93	48.36 ± 7.39	6 (54.5)	6 (54.5)	NR		6 (54.5)	6 (54.5)
Polat (2003) [35]	1	NR	45 ± 1.3	54 ± 1.4	6 (50)	7 (58.33)	NR		NR	
Sefr (2003) [46]	Intraop evaluation	10-15^b^ reverse	53.8 ± 15.04	54.1 ± 14.24	4 (26.66)	3 (20)	NR		8 (53.33)	9 (60)
Eryılmaz (2012) [8]	1	NR	49.40 ± 12.7	51.73 ± 12.5	3 (15)	14 (60.86)	NR		12 (60)	14 (60.86)

*Pts* patients, *h* hour, *d* day, *SD* standard deviation, *VAS* visual analogue scale, *N* number, *LP* low pressure, *S/HP* standard/high-pressure group, *NR* not reported, *NA* not available (data present only in figures not explain in article text), *ref* reference

^a^Trendelenburg position; *ASA* American Society of Anaesthesia, *BMI* body mass index;

^b^Male in total sample size

**Table 4:** Patients characteristics of the included RCTs with more than 2 study groups

Author (year) [refs]	Study arms	Mean age in years (SD)	Male (%)	Mean BMI in kg/m^2^ (SD)	ASA I *N*(% of pts)	Trend^a^	Follow-up duration (days)
Barrio (2017) [49]	LP + moderate- NMB (8 mmHg)	46.97 ± 14.27	9	25.66 ± 3.16	10	25^b^ Reverse Trend in French position	
	LP + deep- NMB (8 mmHg)	51.13 ± 10.13	10	25.67 ± 3.29	10		
	Standard (12 mmHg)	51.43 ± 10.28	11	26.53 ± 2.97	9		
Celik (2010) [50]	Low (8 mmHg)	42.9 ± 10.8	0	72.6 ± 9.2	NR	NR	NR
	Standard (12 mmHg)	43.8 ± 9.9		72.7 ± 9.3			
	High (14 mmHg)	45.3 ± 8.6		73.5 ± 9.8			
Kandil et al. (2010) [51]	Low (8 mmHg)	42.38 ± 10.67 (range 18–61) in all groups	38 (38) in all groups	NR	NR	NR	10
	Median (10 mmHg)						
	Standard (12 mmHg)						
	High (14 mmHg)						
Esmat (2006) [52]	High (14 mmHg)	Median 46.6 (range 24–63)	32	NR	NR	NR	2
	Low (10 mmHg)	Median 47.8 (range 22–65)	37	NR	NR	NR	2
	Low + saline (10 mmHg)	Median 45.8 (range 23–63)	35	NR	NR	NR	2
Umar (2013) [54]	Group 1 (8–10 mmHg)	NR	NR	NR	NR	Reverse Trendlenburg 15^b^	1
	Group 2 (11–13 mmHg)						
	Group 3 (≥ 14 mmHg)						
Topal (2011) [55]	1 (10 mmHg)	42.71 ± 10.12	16 (80)	NR	NR	30^b^ reverse trendelenburg position	1
	2 (13 mmHg)	39.82 ± 11.85	14 (70)				
	3 (16 mmHg)	43.76 ± 9.81	17 (85)				
Celik (2004) [56]	I (8 mmHg)	43 ± 15	3 (15)	NR	NR	NR	1 h
	II (10 mmHg)	46 ± 9	5 (25)				
	III (12 mmHg)	40 ± 12	4 (20)				
	IV (14 mmHg)	43 ± 15	2 (10)				
	V (16 mmHg)	39 ± 13	5 (25)				

*Pts* patients, *N* number, *yrs* years, *BMI* body mass index, *ASA* American Society of Anesthesiologists, *NR* not reported

^a^Trendelenburg position; *NMB* neuromuscular blockade, *LPG* low-pressure group, *HPG* high-pressure group, *SPG* standard-pressure group, *PTC* post tetanic count, *BMI* body mass index

Tables 3, 4, 5, and 6 show the raw data of the included articles regarding length of hospital stay, conversion to open surgery, conversion to higher pressure, operative time, and level of satisfaction.

**Table 5:** Post-operative pain outcomes and analgesic consumption from RCTs included in Metanalysis.

Author (year) [refs]	LOS			Conversion open n of pts			Conversion increased pressure n of pts		Operative time (min)* Mean ± SD			Level of satisfaction	
	LP	S/HP		LP	S/HP		LP	S/HP	LP	S/HP		LP	S/HP
Chock (2006) [15]	NR			0	0		3	0	73.6 ± 16.3	71.0 ± 29.3		9.05 ± 1.00^a^	9.10 ± 1.37^a^
Ekici (2009) [16]	NR			0	0		1	0	55.05 ± 20.19	51.02 ± 17.23		NR	
Ibraehim (2006) [17]	NR			NR			NR		55.7 ± 8.6	51.9 ± 8.3		NR	
Joshipura (2009) [18]	27 ± 2.33 h	43 ± 7.74 h		0	0		4	0	60.35 ± 6.54	61.67 ± 12.83		Vision dissection, space for dissection, and vision, whereas use of suction were felt inadequate by all the surgeons with LPLC as compared with HPLC	
Koc (2005) [19]	NR			(3)^b^			NR		56.7 ± 19.2		59.4 ± 21.7	NR	
Perrakis (2003) [20]	NR			NR			2	0	31.5 ± 14.4	36.25 ± 18.7		NR	
Wallace (1997) [21]	1.5 days (IQR 1–2)	2 days (IQR 2–3)		NS	1		5	0	51.5 ± 6.35	53 ± 6.93		NR	
Zaman (2015) [22]	NR			NR			NR		NR			NR	
Ali (2016) [1]	NR			NR			NR		27.84 ± 6.078	28.51 ± 7.45		NR	
Barczynski (2002) [27]	NR			NR			NR		NR			NR	
Barczynski (2003) [28]	2.05 ± 0.4 days	2.10 ± 0.4 days		0	0		4	1	55.7 ± 8.6	51.9 ± 8.3		QoL at 7th post-op day: 78% LPG vs 89% SPG, *p* < 0.01	
Bhattacharjee (2017) [29]	NR			0	0		0	0	38.5 ± 12.6	38 ± 12.4		Surgeon satisfaction: similar in two groups	
Karagulle (2009) [30]	NR			1^b^	0		NR		55.8 ± 9.1	50.5 ± 12.6		NR	
Kanwer (2009) [31]	NR			0^b^	2^b^		3^b^	0^b^	49.07 ± 5.72	46.43 ± 6.92		NR	
Morino (1998) [32]	NR			NR			NR		NR			NR	
Hasukič (2005) [23]	NR			NR			NR		104 ± 25.04 (Range 60–150)	99.40 ± 29.73 (Range 60–180)		NR	
Donmez (2016) [24]	NR			NR			NR		54 ± 9	57 ± 6		NR	
Filho (2021) [25]	NR			NR			NR		NR			NR	
Dexter (1999) [26]	NR			0^b^		1^b^	2^b^	0^b^	109.25 ± 37.53	118.75 ± 34.64		NR	
Gupta (2013) [36]	NR			0	0		0	0	48.00 ± 7.76	47.25 ± 6.73		NR	
Goel (2019) [37]	NR			1	1		2	0	62.6 ± 4.5	60.45 ± 5.6		NR	
Ko-iam (2016) [38]	1 day 53 (96.4) > 1 day2 (3.6)	1 day 45 (75.0) > 1 day15 (25.0)		(5)	0		NR		56.8 ± 17.6	56.7 ± 16.3		NR	
Mohammadzade (2016) [39]	NR			NR			NR		NR			NR	
Nasajiyan (2014) [40]	NR			NR			NR		121.3 ± 13.4	107.5 ± 10.4		NR	
Singla (2014) [41]	NR			NR			NR		39.16 ± 5.14	39.36 ± 5.43		NR	
Shoar (2015) [42]	NR			NR			NR		53.6 ± 25.1	47.8 ± 16.8		NR	
Torres (2009) [43]	NR			NR			NR		45 MIN			NR	
Yasir (2012) [44]	1.1 ± 0.45 LPG		1.21 ± 0.36 HPG	0			NR		34.38 ± 5.26	31.52 ± 4.68		NR	
Vijayaraghavan (2012) [45]	NR			1	1		NR		60 ± 45–81.25	60 ± 45–80		Visibility 2 (2–2) 3 (2–3) 0.000 Visibility at suction 1 (1–1) 2 (2–3) 0.000 Space for dissection	
Sarli (2000) [47]	1.3		1.4	NR			NR		36.2	39.2		NR	
Sandhu (2008) [48]	1.13 ± 0.38 days		1.29 ± 0.70 days	0	0		2	0	61.32 ± 22.58	62.54 ± 20.30		VAS (1–10), mean ± SD: 3.14 ± 2.20; *p* = 0.07	VAS (1–10), mean ± SD: 4.04 ± 2.06; *p* = 0.07
Neogi (2019) [4]	NR			1	1		8	0	56.4		54	NR	
Basgul (2004) [33]	NR			NR			NR		65.27 ± 5.61	64.27 ± 6.13		NR	
Polat (2003) [35]	NR			NR			NR		70.9 ± 3	66 ± 3.5		NR	
Sefr (2003) [46]	NR			0	0		NR		57.5 ± 23.20	58.6 ± 11.76		NR	
Eryılmaz (2012) [8]	NR			NR			NR		50.2 ± 19.1	58.5 ± 24.5		NR	

*Pts* patients, *h* hour, *d* day, *SD* standard deviation, *VAS* visual analogue scale, *N* number, *LP* low pressure, *S/HP* standard/high-pressure group, *PTC* post tetanic count, *NR* not reported, *ref* reference, *LOS* length of stay

^a^Patient satisfaction was assessed in visual analogue scale on post-operative day 3

^b^Patients converted and excluded from the studies

**Table 6:** Outcomes characteristics of RCTs with more than 2 study groups

Author (year) [refs]	Study arms	Conversion open	Conversion increased pressure	Operative time (min)*	LOS	Level of satisfaction
Barrio (2017) [49]	LP + moderate- NMB (8 mmHg)	0	1	42.76 ± 15.17	NR	NR
	LP + deep- NMB (8 mmHg)	0	4	44 ± 13.18		
	Standard (12 mmHg)	0	0	42.2 ± 11.39		
Celik (2010) [50]	Low (8 mmHg)	0	0	31.3 ± 9	NR	NR
	Standard (12 mmHg)	0	0	29.2 ± 5.5 SPG vs HPG p < 0.05		
	High (14 mmHg)	NR	NR	36.17 ± 9.2 SPG vs HPG p < 0.05		
Kandil et al. (2010) [51]	Low (8 mmHg)	0		36 ± 9.9	NR	NR
	Median (10 mmHg)					
	Standard (12 mmHg)					
	High (14 mmHg)					
Esmat (2006) [52]	High (14 mmHg)	6 pts	11 pts low to high	Mean 43.7 (range 29–57)	1.4 (1–3)	NR
	Low (10 mmHg)			Mean 45.2 (range 25–62)	1.7 (1–3)	NR
	Low + saline (10 mmHg)			Mean 54.4 (range 42–68)	1.6 (1–3)	NR
Umar (2013) [54]	Group 1 (8–10 mmHg)	NR	NR	NR	NR	NR
	Group 2 (11–13 mmHg)					
	Group 3 (≥ 14 mmHg)					
Topal (2011) [55]	1 (10 mmHg)	NR	NR	42.12 ± 11.63	NR	NR
	2 (13 mmHg)			41.84 ± 9.12		
	3 (16 mmHg)			46.36 ± 10.34		
Celik (2004) [56]	I (8 mmHg)	NR	NR	65 ± 11	NR	NR
	II (10 mmHg)			56 ± 11		
	III (12 mmHg)			58 ± 15		
	IV (14 mmHg)			64 ± 12		
	V (16 mmHg)			55 ± 9		
Gin (2021) [53]	Low (8 mmHg)	NR	More patients in the LPLC group required a pressure increase to a higher pressure than in the SPLC group (15 pts in LPG (29%) vs 4 pts in HPG (8%), p = 0.010)	62.5 median (IQR 47, 77)	1 median (IQR 0, 2)	22% surgeon operate with LP vs 65% prefer HP
	Standard (12 mmHg)			67 (49, 78.5)	1 median (IQR 0, 2)	
	High (14 mmHg)			76.5 (55.5, 104)	1 median (IQR 0, 2)	

*Pts* patients, *h* hour, *d* day, *SD* standard deviation, *VAS* visual analogue scale, *N* number, *LP* low pressure, *S/HP* standard/high-pressure group, *PTC* post-tetanic count, *NR* not reported, *ref* reference, *LOS* length of stay, *NMB* neuromuscular blockade, *PTC* post-tetanic count

Tables 3, 4, 5, and 6 show the results relating to post-operative pain. In all included articles pain was evaluated by a Visual Analogue Scale (VAS). The time of pain evaluation ranges between one hour after surgery and three days after surgery.

Table 7 shows the complications occurred. Overall 96 and 74 intra- and post-operative complications were observed among patients who underwent cholecystectomy with low pressure and with high pressure, respectively.

**Table 7:** Post-operative pain outcomes from RCTs included in Metanalysis

Author (year) [ref]	Post-op pain VAS at 1 h			Post-op pain VAS at 1 d							Post-op pain VAS at 2 d				Post-op pain VAS at 3 d				Post-op pain VAS at 8 h Mean ± SD					Post-op pain VAS at 2 h Mean ± SD					Post-op pain VAS at 3 h Mean ± SD		Post-op pain VAS at 4 h Mean ± SD					Post-op pain VAS overall Mean ± SD			
	LP		S/H P	LP	S/HP						LP	S/HP			LP	S/H P			LP	S/ HP				LP	S/H P				LP	S/HP	LP	S/HP				LP	S/HP		
Chock (2006) [15]	NR			2.85 ± 2.03	3.05 ± 1.70						NR	NR			1.75 ± 2.15	0.70 ± 1.13			NR	NR				NR					NR		NR					NR			
Ekici (2009) [16]	NR			NR							NR				NR				NR					NR					NR		NR					NR			
Ibraehim (2006) [17]	NS			NR							NR				NR				NR					5.0 ± 1 .886	7.4 ± 1 .17				NR (P < = 0.05)		NR					NR			
Joshipura (2009) [18]	NR			10.71	22.5						NR				NR				16.2 1	30				NR					NR		24.29	38.75				NR			
Koc (2005) [19]	NR			1.3 ± 0.9	1.7 ± 1.0						NR				NR				NR					NR					NR		NR					NR			
Perrakis (2003) [20]	NR			NR															NR					NR					NR		NR					NR			
Wallace (1997) [21]	NR			45.5 ± 9.8°	85 ± 22.49°						NR	NR			28 ± 10.9 At 6d	41 ± 14.45 At 6d			59.2 ± 8.3 6 H°	88 ± 7.8 6 H°				NR					NR		NR					NR			
Zaman (2015) [22]	0.92 ± 3.19 Over all		5.72 ± 8.59 Over all	NR							NR				NR				NR					NR					NR		NR					NR			
Ali (2016) [1]	NR			NR							NR				27.84 ± 6			28.5 1 ± 7	NR					NR					NR		NR					0.28 ± 0.90			1.31 ± 2 .38
Barczynski (2002) [27]	NR			NR							NR				NR				NR					NR					NR		NR					NR			
Barczynski (2003) [28]	NR			31.79 ± 5.17			36.54 ± 6.62				29.94 ± 4.74			41.10 ± 11.17	28.82 ± 5.07		39.32 ± 7.71		28.54 ± 7.23		32.93 ± 9.15			NR					NR		27.62 ± 7.32			31.78 ± 9.21		NR			
Bhattacharjee (2017) [29]	NA			NR							NR				NR				NA					NR					NR		NA					NR			
Karagulle (2009) [30]	NR			NR							NR				NR				NR					NR					NR		NR					NR			
Kanwer (2009) [31]	NR			4.60 ± 0.81					5.2 ± 0.8		NR				NR				62.2 ± 11.7			59.1 ± 18.0		54.2 ± 8.5				62.2 ± 12	NR		NR					NR			
Morino (1998) [32]	NR			NR							NR				NR				NR					at 6 h					at 6 h		At 12 h					At 12 h			
Hasukič (2005) [23]	NR			NR							NR				NR				NR					NR					NR		NR					NR			
Donmez (2016) [24]	NR			NR							NR				NR				NR					NR					NR		NR					NR			
Filho (2021) [25]	NR			NR							NR				NR				NR					NR					NR		NR					NR			
Dexter (1999) [26]	NR			NR							NR				NR				NR					NR					NR		NR					NR			
Gupta (2013) [36]	NR			NR							NR				NR				NR					NR					NR		NR					NR			
Goel (2019) [37]	NR			2.67 ± 1.20						4.01 ± 0.87	2.14 ± 1.11		2.65 ± 1.53		NR				NR					0.45 ± 0.30 At 12 h			2.12 ± 0.54 At 12 h		NR		NR					NR			
Ko-iam (2016) [38]	NA			NA							NA				NA				NA					NA					NA		NA					NA			
Mohammadzade (2016) [39]	NA			NA							NA				NA				NA					NA					NA		NA					NA			
Nasajiyan (2014) [40]	NR			NR							NR				NR				NR					NR					NR		NR					NR			
Singla (2014) [41]	0.14 ± 0.48	0.46 ± 0.72		0.08 ± 0.27				1 ± 1.56			NR				NR				NR					0.28 ± 0.97		1.26 ± 1.9			NR		0.36 ± 1.24		1.44 ± 2.19			1.42 ± 4.88		7.88 ± 11.76	
Shoar (2015) [42]	NR			NR							NR				NR				NR					NR					NR		NR					NR			
Torres (2009) [43]	NR			NR							NR				NR				NR					NR					NR		NR					NR			
Yasir (2012) [44]	NR			NR							NR				NR				NR					NR					NR		NR					NR			
Vijayaraghavan (2012) [45]	NR			1.5 ± 0.57		2.75 ± 0.14					NR				NR				2 ± 0.61				3 ± 0.61	NR					NR		2.18 ± 0.41				4 ± 0.61	NR			
Sarli (2000) [47]	NR			NR							NR				NR				NR					NR					NR		NR					NR			
Sandhu (2008) [48]	NR			NR							NR				NR				NR					NR					NR		NR					3.14 ± 2.20	4.04 ± 2.06		
Neogi (2019) [4]	NR			NR							NR				NR				NR					NR					NR		NR					NR			
Basgul (2004) [33]	NR			NR							NR				NR				NR					NR					NR		NR					NR			
Polat (2003) [35]	NR			NR							NR				NR				NR					NR					NR		NR					NR			
Sefr (2003) [46]	NR			NR							NR				NR				NR					NR					NR		NR					NR			
Eryılmaz (2012) [8]	NR			NR							NR				NR				NR					NR					NR		NR					NR			
Neogi (2019) [4]	NR			NR							NR				NR				NR					NR					NR		NR					NR			
Basgul (2004) [33]	NR			NR							NR				NR				NR					NR					NR		NR					NR			
Polat (2003) [35]	NR			NR							NR				NR				NR					NR					NR		NR					NR			
Sefr 2003) [46]	NR			NR							NR				NR				NR					NR					NR		NR					NR			
Eryılmaz (2012) [8]	NR			NR							NR				NR				NR					NR					NR		NR					NR			

*Pts* patients, *h* hour, *d* day, *SD* standard deviation, *VAS* visual analogue scale, *N* number, *LP* low pressure, *S/HP* standard/high-pressure group, *PTC* post tetanic count, *NR* not reported, *NA* not available (data present only in figures not explain in article text), *ref* reference

^a^On movement

## Discussion

Laparoscopic surgery has increased in popularity in recent years due to a reduced operative stress response and improved clinical outcomes including reduced operation time, bleeding, opioid requirement, and reduced LOS when compared to open surgery [60].

The creation of pneumoperitoneum may be overlooked or not considered a significant operative factor, however, it constitutes the first step of every laparoscopic procedure and should be given due consideration.

In this systematic review of the available literature on the topic, we found out that lowering the pneumoperitoneum pressure has a positive impact on post-operative pain, while may be linked to longer operative time when considering elective laparoscopic cholecystectomy.

Traditionally, the standard intra-abdominal pressure used was around 15 mmHg [3]; although laparoscopic surgery is labelled a minimally invasive procedure, such pressures may lead to a disruption in mechanical and biochemical balance.

The cardiovascular and pulmonary systems are the most affected by increased intra-abdominal pressure as demonstrated in several published studies [61–64]. Although these cardiorespiratory changes may be tolerated by healthy adults with adequate cardiopulmonary reserve, when these reserves are compromised, the use of laparoscopy is limited [12]. As laparoscopic procedures become standardized, the question arises as to the optimum maintenance pressure for pneumoperitoneum.

International guidelines recommend the use of ‘the lowest intra-abdominal pressure allowing adequate exposure of the operative field rather than a routine pressure” [64]. In a previous meta-analysis, overall quality of evidence for advantages of low-pressure PP compared to high-pressure PP was evaluated [65]. The meta-analysis took into consideration all published papers where a low-pressure (LP) peritoneum was used. The authors concluded that the main impact of the use of low-pressure pneumoperitoneum is on post-operative pain and analgesic consumption, but the safety profile of LP must be better defined, as the analysis of the existing literature could only produce a low-to-moderate level of evidence.

In this study, we chose to consider elective laparoscopic cholecystectomy only, as the index procedure for the meta-analysis for two main reasons: firstly, to reduce bias linked to outcomes related to the complexity of laparoscopic procedures and secondly, because the elective laparoscopic cholecystectomy is considered a cornerstone procedure for the minimally invasive surgeon.

A Cochrane review already exists on this topic, and the primary conclusion was that although laparoscopic cholecystectomy can be completed successfully using low pressure in approximately 90% of people undergoing laparoscopic cholecystectomy, no conclusive evidence exists to support its utilization of LP in healthy low anaesthetic risk patients and that the safety must be better defined. As a result of this, the authors did not recommend LP pneumoperitoneum unless future trials demonstrate a clinical benefit. Although, a significative reduction in post-operative shoulder pain was demonstrated, its influence on other considered parameters was either inconclusive or not significant. In conclusion, though lowering intra-abdominal pressure may decrease the associated detrimental effects of standard/high-pressure pneumoperitoneum, the safety of low-pressure pneumoperitoneum has not been fully defined.

In our analysis, the pressures reported as low in the considered studied ranged from 6 to 10 mmHG. While in the standard/high-pressure groups, 12 to 15 mmHg pressures were applied.

Regarding post-operative pain, the time-frame considered in the included studies was highly variable. However, generally, patients in the low-pressure group reported lower VAS if compared with patients in the standard-pressure group. This difference was less significant in the first and second post-operative days and was not reported 3 days from the operation. Nevertheless, the evaluation of shoulder pain was reported in 12 studies and patients in the low-pressure group reported significantly lower rates of post-operative shoulder pain compared with patients in the standard-pressure group. These findings were associated with a significantly lower analgesic consumption reported at any time by patients in the low-pressure group.

Pain after laparoscopic procedures can be divided into three components: referred shoulder pain, superficial or incisional wound pain, and deep intra-abdominal pain [66]. The different types of pain may correspond to different etiologies. Referred pain is most often attributed to CO2-induced diaphragm and/or phrenic nerve irritation causing referred pain to the C4 dermatome, stretching of the diaphragm, and/or residual pockets of gas in the abdominal cavity [67, 68]. Deep intra-abdominal pain is mainly caused by bowel traction, stretch of the abdominal wall, and compression of intra-abdominal organs. However, according to the results of our review, such symptoms could be attributable to the pressure of the pneumoperitoneum.

Unlike the pre-existing review, we found that a lower pressure may significantly increase the operative time. Only 8 studies reported shorter operative times in the LPLC group and this difference was never significant, compared with the remaining 36 studies, where the operative time in the LPLC groups was always, and in many cases, significantly [51], higher.

A prolonged operative time was reported to be a consequence of the surgeon’s reduced visibility [40]. The reported reduced visibility was not, however, associated with an increased rate of intra-operative complications or conversion rate.

The effect of a prolonged operative time with a low-pressure peritoneum on clinical outcomes was not deducible from the included studies.

When considered, cardiac and pulmonary function did not appear to differ between the included groups. Ekici et al. [16] report on the effect of high-pressure laparoscopic cholecystectomy (HPLC) on QT length. They report a significant increase in the QT dispersion (QTd) and was associated with QT dispersion (QTcd) in the HPLC group. Additionally, there was a temporary increase in HR, which was significantly higher in the HPLC group. Such increases in QTd and QTc are associated with increased risk of arrhythmias and cardiac events. Similarly, the Umar et al. paper reports a significant increase in mean HR, SP, and MAP during insufflation, at exsufflation and at 10, 20, and 30 min after exsufflation in the HPLC group. It was concluded that high-pressure pneumoperitoneum resulted in greater changes in haemodynamic parameters as well as peritoneal CO2 absorption.

The majority of the participants in the trials reviewed were low anaesthetic risk patients undergoing elective laparoscopic cholecystectomy. Therefore, the findings of this review are applicable only to a similar group of patients.

Interestingly, we observed that, unlike previous reviews, most of the included trials were assessed as having a low risk of bias.

As compared with many other surgical trials, the pneumoperitoneum pressure offers an easily measurable factor, meaning it is possible to perform large scale randomized trials, which has allowed us to draw conclusive results from the use of LPLC.

Potential biases are mainly linked to the difficulties associated with blinding the operators. The quality of the evidence is moderate to high for conversion and post-operative pain, respectively.

## Supplementary Information

Below is the link to the electronic supplementary material.

Supplementary file1 (JPEG 151 kb)

Supplementary file2 (JPEG 441 kb)

Supplementary file3 (JPEG 185 kb)

Supplementary file4 (JPEG 131 kb)

Supplementary file5 (JPEG 141 kb)

Supplementary file6 (JPEG 161 kb)

Supplementary file7 (JPEG 148 kb)

Supplementary file8 (JPEG 196 kb)

Supplementary file9 (JPEG 130 kb)

Supplementary file10 (JPEG 141 kb)

Supplementary file11 (JPEG 142 kb)

Supplementary file12 (JPEG 225 kb)

Supplementary file13 (JPEG 163 kb)

Supplementary file14 (JPEG 142 kb)

Supplementary file15 (JPEG 153 kb)

Supplementary file16 (JPEG 220 kb)

Supplementary file17 (JPEG 125 kb)

Supplementary file18 (JPEG 181 kb)

Supplementary file19 (DOCX 30 kb)

Supplementary file20 (DOC 86 kb)

## References

[1] Ali IS, Shah MF, Faraz A, Khan M (2016). Effect of intra-abdominal pressure on post-laparoscopic cholecystectomy shoulder tip pain: a randomized control trial. JPMA J Pak Med Assoc.

[2] Shi HY, Lee HH, Tsai JT, Ho WH, Chen CF, Lee KT (2012). Comparisons of prediction models of quality of life after laproscopic cholecystectomy: a longitudinal prospective study. PLoS ONE.

[3] Russell RC (1993). General surgery: biliary surgery. BMJ (Clinical Research Ed).

[4] Neogi P, Kumar P, Kumar S (2020). Low-pressure pneumoperitoneum in laparoscopic cholecystectomy: a randomized controlled trial. Surg Laparosc Endosc Percutan Techn.

[5] Korkmaz A, Alkiş M, Hamamci O (2002). Hemodynamic changes during gaseous and gasless laparoscopic cholecystectomy. Surg Today.

[6] Wright DM, Serpell MG, Baxter JN (1995). Effect of extraperitoneal carbon dioxide insufflation on intraoperative blood gas and hemodynamic changes. Surg Endosc.

[7] Schietroma M, Carlei F, Cecilia EM (2013). A prospective randomized study of systemic inflammation and immune response after laparoscopic Nissen fundoplication performed with standard and low-pressure pneumoperitoneum. Surg Laparosc Endosc Percutan Tech.

[8] Eryilmaz HB, Memiş D, Sezer A (2012). The effects of different insufflation pressures on liver functions assessed with LiMON on patients undergoing laparoscopic cholecystectomy. Sci World J.

[9] Suginami R, Taniguchi F, Suginami H (2009). Prevention of postlaparoscopic shoulder pain by forced evacuation of residual CO2. JSLS.

[10] Jian ZF, Jie Li, Ze LU (2009) Effect of implanting fibrin sealant with J Pak Med Assoc (Suppl. 3) 2nd Annual Surgical Meeting 2016 ropivacaine on pain after laproscopic cholecystectomy. World J Gastroenterol 15:5851-5854 10.3748/wjg.15.5851 PMC2791280 19998508

[11] Sandhu T, Yamada S, Ariyakachon V, Chakrabandhu T, Chongruksut W, Ko-iam W (2009). Low-pressure pneumoperitoneum versus standard pneumoperitoneum in laparoscopic cholecystectomy, a prospective randomized clinical trial. Surg Endosc.

[12] Gurusamy KS, Vaughan J, Davidson BR (2014). Low pressure versus standard pressure pneumoperitoneum in laparoscopic cholecystectomy. Cochrane Database Syst Rev.

[13] Page MJ, McKenzie JE, Bossuyt PM, Boutron I, Hoffmann TC, Mulrow CD (2021). The PRISMA 2020 statement: an updated guideline for reporting systematic reviews. BMJ.

[14] Higgins JPT, Thomas J, Chandler J, Cumpston M, Li T, Page MJ, Welch VA (eds) (2021) Cochrane Handbook for Systematic Reviews of Interventions version 6.2 (updated February 2021). Cochrane. http://www.training.cochrane.org/handbook 10.1002/14651858.ED000142 PMC10284251 31643080

[15] Chok KS, Yuen WK, Lau H (2006). Prospective randomized trial on low-pressure versus standard-pressure pneumoperitoneum in outpatient laparoscopic cholecystectomy. Surg Laparosc Endosc Percutan Tech.

[16] Ekici Y, Bozbas H, Karakayali F (2009). Effect of different intra-abdominal pressure levels on QT dispersion in patients undergoing laparoscopic cholecystectomy. Surg Endosc.

[17] Ibraheim OA, Samarkandi AH, Alshehry H (2006). Lactate and acid base changes during laparoscopic cholecystectomy. Middle East J Anaesthesiol.

[18] Joshipura VP, Haribhakti SP, Patel NR (2009). A prospective randomized, controlled study comparing low pressure versus high pressure pneumoperitoneum during laparoscopic cholecystectomy. Surg Laparosc Endosc Percutan Tech.

[19] Koc M, Ertan T, Tez M (2005). Randomized, prospective comparison of postoperative pain in low- versus high-pressure pneumoperitoneum. ANZ J Surg.

[20] Perrakis E, Vezakis A, Velimezis G (2003). Randomized comparison between different insufflation pressures for laparoscopic cholecystectomy. Surg Laparosc Endosc Percutan Tech.

[21] Wallace DH, Serpell MG, Baxter JN (1997). Randomized trial of different insufflation pressures for laparoscopic cholecystectomy. Br J Surg.

[22] Zaman M, Chowdhary K, Goyal P (2015). Prosepecive randomized trial of low pressur prenumoperitoneum for reduction of shoulder tip pain following laparoscopica cholecystectomy: a comparative study. World J Laparosc Surg.

[23] Hasukic S (2005). Postoperative changes in liver function tests Randomized comparison of low- and high-pressure laparoscopic cholecystectomy. Surg Endosc.

[24] Donmez T, Uzman S, Yildirim D (2016). Is there any effect of pneumoperitoneum pressure on coagulation and fibrinolysis during laparoscopic cholecystectomy?. PeerJ.

[25] Marton Filho MA, Alves RL, Nascimento PD (2021). Effects of pneumoperitoneum on kidney injury biomarkers: A randomized clinical trial. PLoS ONE.

[26] Dexter SPL, Vucevic M, Gibson J (1999). Hemodynamic consequences of high- and low-pressure capnoperitoneum during laparoscopic cholecystectomy. Surg Endosc.

[27] Barczynski M, Herman RM (2002). Influence of different pressures of pneumoperitoneum on the autonomic system function during laparoscopy. Folia Med Cracov.

[28] Barczynski M, Herman RM (2003). A prospective randomized trial on comparison of low-pressure (LP) and standard-pressure (SP) pneumoperitoneum for laparoscopic cholecystectomy. Surg Endosc.

[29] Bhattacharjee HK, Jalaludeen A, Bansal V (2017). Impact of standard-pressure and low-pressure pneumoperitoneum on shoulder pain following laparoscopic cholecystectomy: a randomised controlled trial. Surg Endosc.

[30] Karagulle E, Turk E, Dogan R (2009). Effects of the application of intra-abdominal low pressure on laparoscopic cholecystectomy on acid-base equilibrium. Int Surg.

[31] Kanwer DB, Kaman K, Nedounsejiane M (2009). Comparative study of low pressure versus standard pressure pneumoperitoneum in laparoscopic cholecystectomy–a randomised controlled trial. Trop Gastroenterol.

[32] Morino M, Giraudo G, Festa V (1998). Alterations in hepatic function during laparoscopic surgery. An experimental clinical study. Surg Endosc.

[33] Basgul E, Bahadir B, Celiker V, Karagoz AH, Hamaloglu E, Aypar U (2004). Effects of low and high intra-abdominal pressure on immune response in laparoscopic cholecystectomy. Saudi Med J.

[34] Eryılmaz HB, Memiş D, Sezer A, Inal MT (2012). The effects of different insufflation pressures on liver functions assessed with LiMON on patients undergoing laparoscopic cholecystectomy. Sci World J.

[35] Polat C, Yilmaz S, Serteser M, Koken T, Kahraman A, Dilek ON (2003). The effect of different intraabdominal pressures on lipid peroxidation and protein oxidation status during laparoscopic cholecystectomy. Surg Endosc.

[36] Gupta R, Kaman L, Dahiya D, Gupta N, Singh R (2013). Effects of varying intraperitoneal pressure on liver function tests during laparoscopic cholecystectomy. J Laparoendosc Adv Surg Tech A.

[37] Goel A, Gupta S, Bhagat TS, Garg P (2019). Comparative analysis of hemodynamic changes and shoulder tip pain under standard pressure versus low-pressure pneumoperitoneum in laparoscopic cholecystectomy. Eur J Hepatogastroenterol.

[38] Ko-Iam W, Paiboonworachat S, Pongchairerks P, Junrungsee S, Sandhu T (2016). Combination of etoricoxib and low-pressure pneumoperitoneum versus standard treatment for the management of pain after laparoscopic cholecystectomy: a randomized controlled trial. Surg Endosc.

[39] Mohammadzade AR, Esmaili F (2018). Comparing hemodynamic symptoms and the level of abdominal pain in high- versus low-pressure carbon dioxide in patients undergoing laparoscopic cholecystectomy. Indian J Surg.

[40] Nasajiyan N, Javaherfourosh F, Ghomeishi A, Akhondzadeh R, Pazyar F, Hamoonpou N (2014). Comparison of low and standard pressure gas injection at abdominal cavity on postoperative nausea and vomiting in laparoscopic cholecystectomy. Pak J Med Sci.

[41] Singla S, Mittal G, Raghav S (2014). Pain management after laparoscopic cholecystectomy-a randomized prospective trial of low pressure and standard pressure pneumoperitoneum. J Clin Diagn Res.

[42] Shoar S, Naderan M, Ebrahimpour H (2016). A prospective double-blinded randomized controlled trial comparing systemic stress response in Laparoascopic cholecystectomy between low-pressure and standard-pressure pneumoperitoneum. Int J Surg.

[43] Torres K, Torres A, Staskiewicz GJ (2009). A comparative study of angiogenic and cytokine responses after laparoscopic cholecystectomy performed with standard- and low-pressure pneumoperitoneum. Surg Endosc.

[44] Yasir M, Mehta KS, Hussain Banday V (2012). Evaluation of post operative shoulder tip pain in low pressure versus standard pressure pneumoperitoneum during laparoscopic cholecystectomy. Surgeon.

[45] Vijayaraghavan N, Sistla SC, Kundra P (2014). Comparison of standard-pressure and low-pressure pneumoperitoneum in laparoscopic cholecystectomy: a double blinded randomized controlled study. Surg Laparosc Endosc Percutan Tech.

[46] Sefr R, Puszkailer K, Jagos F (2003). Randomized trial of different intraabdominal pressures and acid-base balance alterations during laparoscopic cholecystectomy. Surg Endosc.

[47] Sarli L, Costi R, Sansebastiano S (2000). Prospective randomized trial of low-pressure pneumoperitoneum for reduction of shoulder-tip pain following laparoscopy. Br J Surg.

[48] Sandhu T, Yamanda S, Ariyakachon V (2009). Low-pressure pneumoperitoneum versus standard pneumoperitoneum in laparoscopic cholecystectomy, a prospective randomized clinical trial. Surg Endosc.

[49] Barrio J, Errando CL, Garcia-Ramon J (2017). Influence of depth of neuromuscular blockade on surgical conditions during low-pressure pneumoperitoneum laparoscopic cholecystectomy: A randomized blinded study. J Clin Anesth.

[50] Celik AS, Frat N, Celebi F (2010). Laparoscopic cholecystectomy and postoperative pain: is it affected by intra-abdominal pressure?. Surg Laparosc Endosc Percutan Tech.

[51] Kandil TS, Hefnawy EE (2010). Shoulder pain following laparoscopic cholecystectomy: factors affecting the incidence and severity. J Laparoendosc Adv Surg Tech A.

[52] Esmat ME, Magdy MA, Magid MA (2006). Combined low pressure pneumoperitoneum and intraperitoneal infusion of normal saline for reducing shoulder tip pain following laparoscopic cholecystectomy. World J Surg.

[53] Gin E, Lowen D, Tacey M, Hodgson R (2021). Reduced laparoscopic intra-abdominal pressure during laparoscopic cholecystectomy and its effect on post-operative pain: a double-blinded randomised control trial. J Gastrointest Surg.

[54] Umar A, Mehta KS, Mehta N (2013). Evaluation of hemodynamic changes using different intra-abdominal pressures for laparoscopic cholecystectomy. Indian J Surg.

[55] Topal A, Celik JB, Tekin A, Yüceaktaş A, Otelcioğlu S (2011). The effects of 3 different intra-abdominal pressures on the thromboelastographic profile during laparoscopic cholecystectomy. Surg Laparosc Endosc Percutan Tech.

[56] Celik V, Salihoglu Z, Demiroluk S, Unal E, Yavuz N, Karaca S, Carkman S, Demiroluk O (2004). Effect of intra-abdominal pressure level on gastric intramucosal pH during pneumoperitoneum. Surg Laparosc Endosc Percutan Tech.

[57] Hozo SP, Djulbegovic B, Hozo I (2005). Estimating the mean and variance from the median, range, and the size of a sample. BMC Med Res Methodol.

[58] Der Simonian R, Laird N (2015). Meta-analysis in clinical trials revisited. Contemp Clin Trials.

[59] GRADEpro GDT: GRADEpro Guideline Development Tool [Software]. McMaster University, 2020 (developed by Evidence Prime, Inc.). https://gradepro.org/cite/gradepro.org

[60] Kim JE, Min SK, Ha E, Lee D, Kim JY, Kwak HJ (2021). Effects of deep neuromuscular block with low-pressure pneumoperitoneum on respiratory mechanics and biotrauma in a steep Trendelenburg position. Sci Rep.

[61] Henny CP, Hofland J (2005). Laparoscopic surgery: pitfalls due to anesthesia, positioning, and pneumoperitoneum. Surg Endosc.

[62] Alijani A, Hanna GB, Cuschieri A (2004). Abdominal wall lift versus positive-pressure capnoperitoneum for laparoscopic cholecystectomy - randomized controlled trial. Ann Surg.

[63] Galizia G, Prizio G, Lieto E, Castellano P, Pelosio L, Imperatore V (2001). Hemodynamic and pulmonary changes during open, carbon dioxide pneumoperitoneum and abdominal wall-lifting cholecystectomy. A prospective, randomized study. Surg Endosc.

[64] Neudecker J, Sauerland S, Neugebauer E, Bergamaschi R, Bonjer HJ, Cuschieri A (2002). The European Association for Endoscopic Surgery clinical practice guideline on the pneumoperitoneum for laparoscopic surgery. Surg Endosc.

[65] Özdemir-van Brunschot DM, van Laarhoven KC, Scheffer GJ, Pouwels S, Wever KE, Warlé MC (2016). What is the evidence for the use of low-pressure pneumoperitoneum? A systematic review. Surg Endosc.

[66] Bisgaard T (2001). Characteristics and prediction of early pain after laparoscopic cholecystectomy. Pain.

[67] Donatsky AM, Bjerrum F, Gogenur I (2013). Surgical techniques to minimize shoulder pain after laparoscopic cholecystectomy. A systematic review. Surg Endosc.

[68] Tsimoyiannis EC (1998). Intraperitoneal normal saline infusion for postoperative pain after laparoscopic cholecystectomy. World J Surg.

