# Supplementary figures and images for: Low-pressure versus standard-pressure pneumoperitoneum in laparoscopic cholecystectomy: a systematic review and meta-analysis of randomized controlled trials
Source: Surg Endosc. 2022 Apr 18;36(10):7092–113. doi: 10.1007/s00464-022-09201-1 (PMC9485078; doi:10.1007/s00464-022-09201-1)

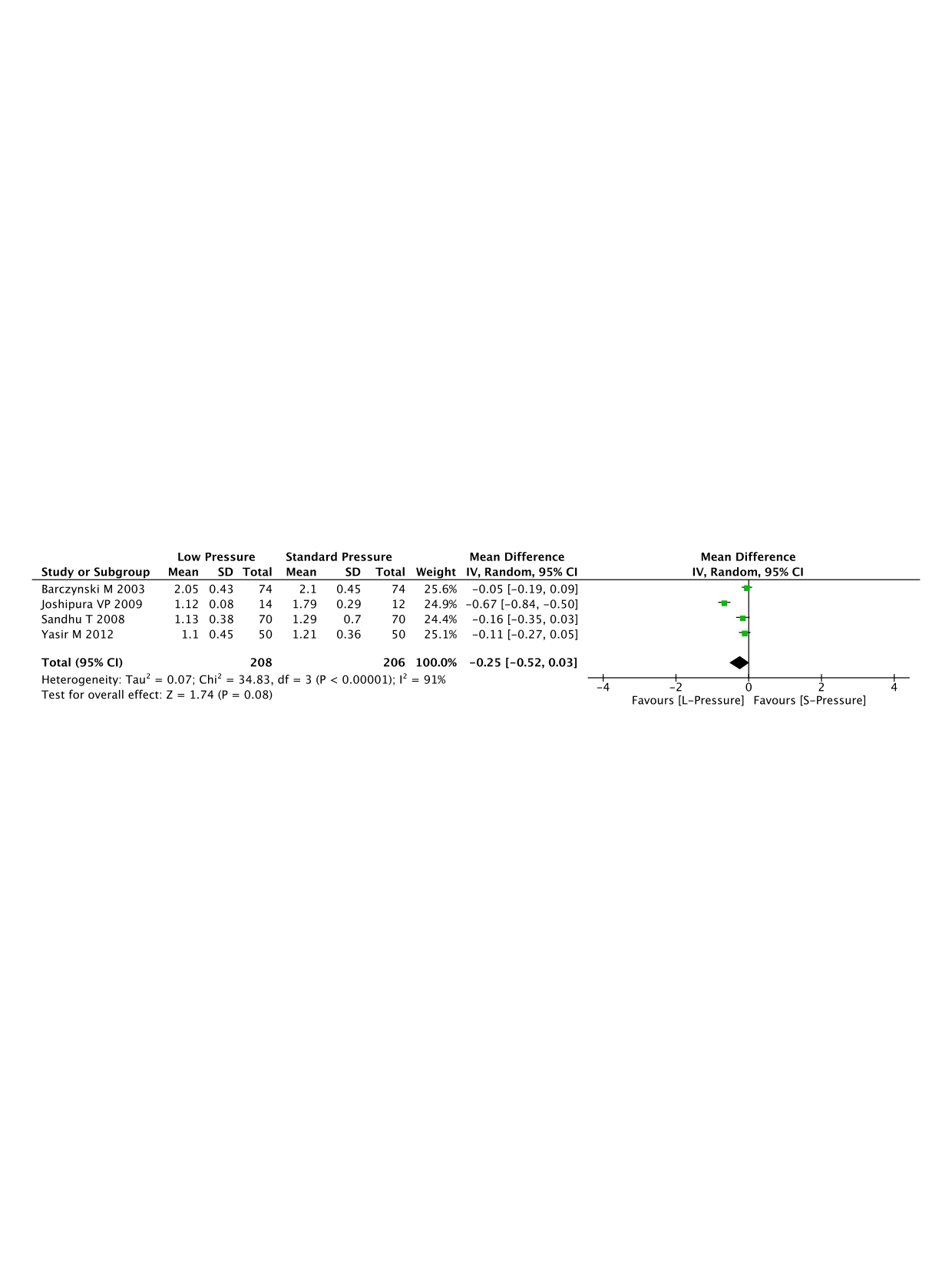

Supplement: Supplementary file 1 — Supplementary file1 (JPEG 151 kb) [file 464_2022_9201_MOESM1_ESM.jpeg]

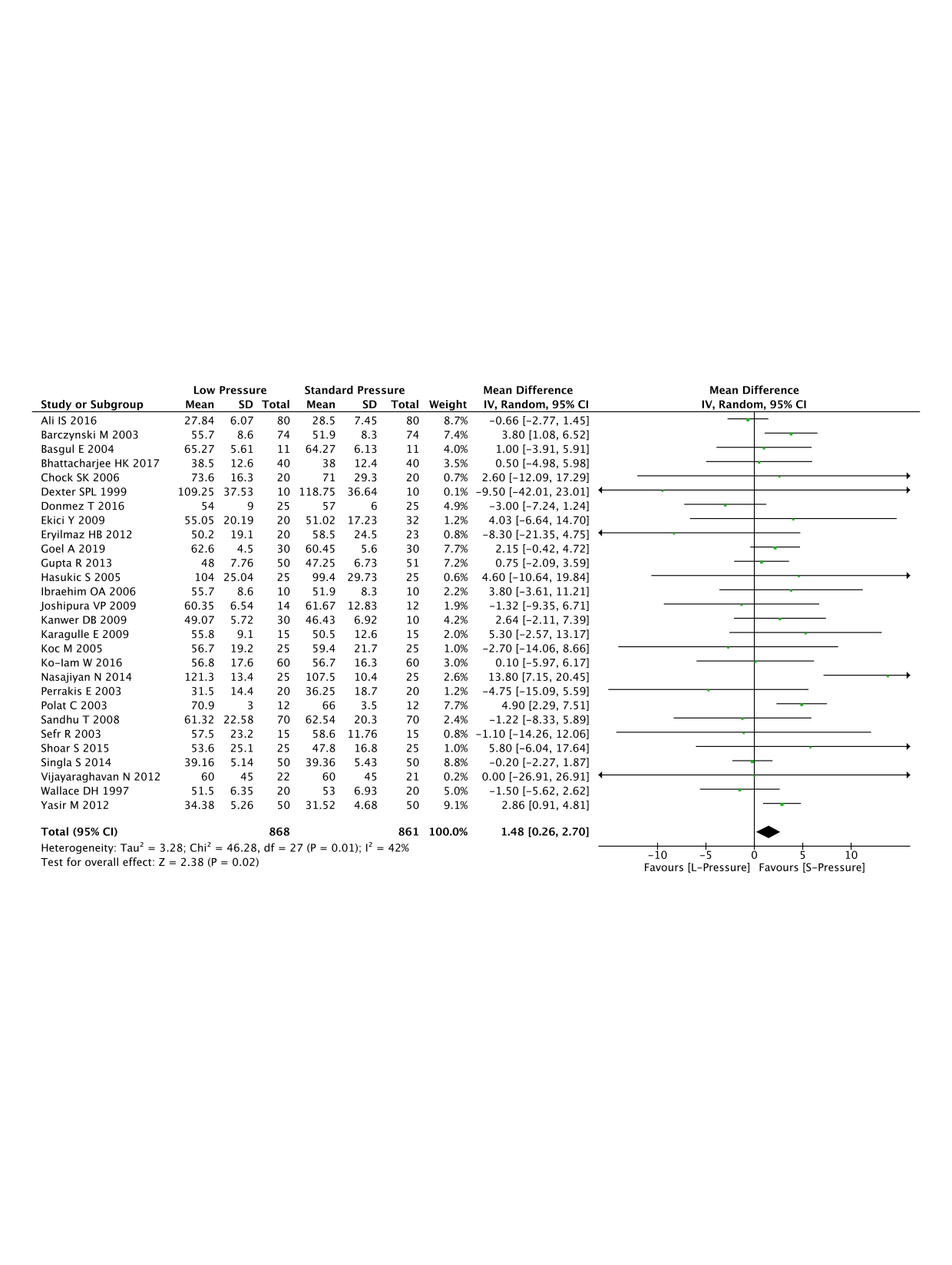

Supplement: Supplementary file 2 — Supplementary file2 (JPEG 441 kb) [file 464_2022_9201_MOESM2_ESM.jpeg]

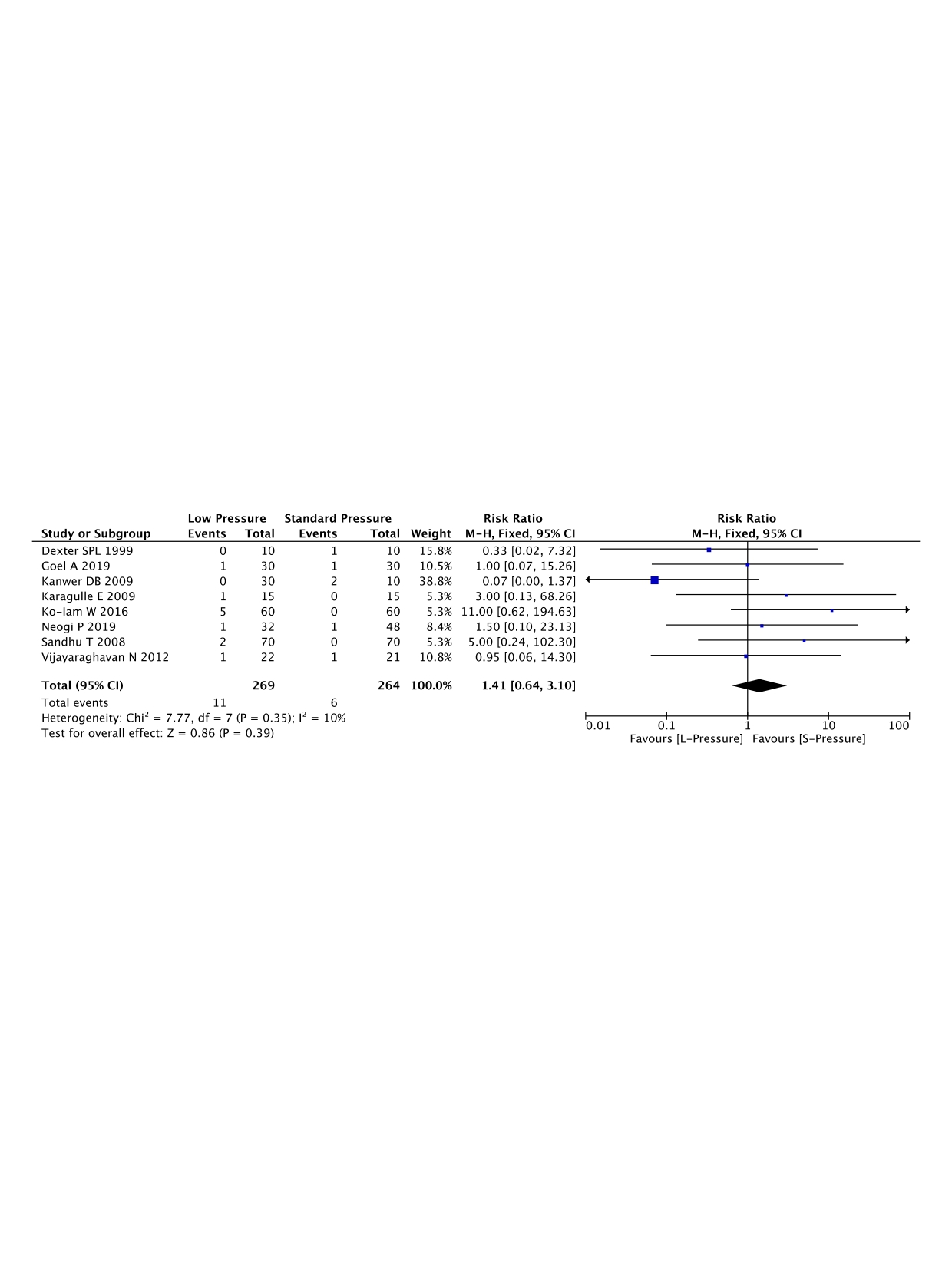

Supplement: Supplementary file 3 — Supplementary file3 (JPEG 185 kb) [file 464_2022_9201_MOESM3_ESM.jpeg]

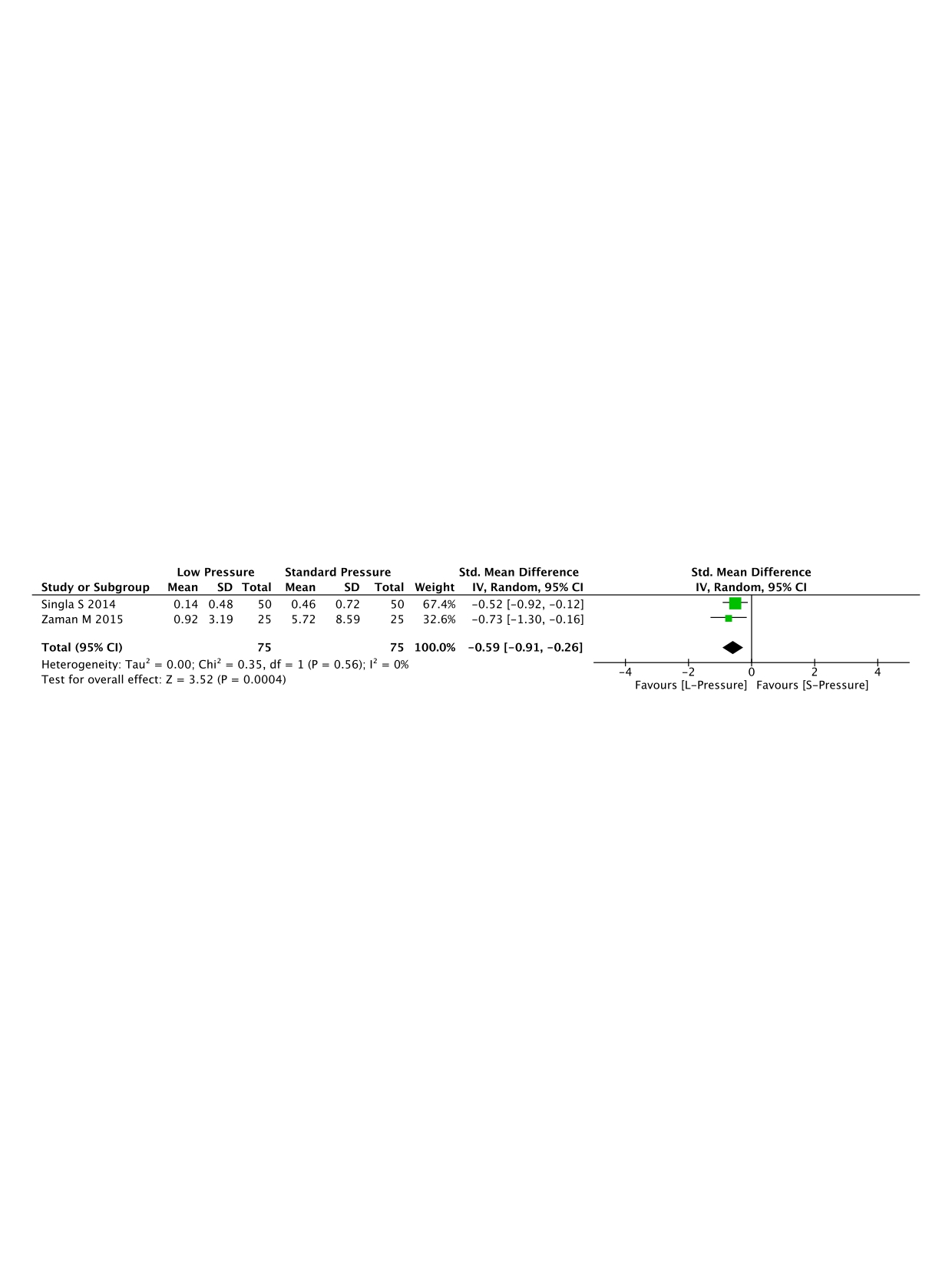

Supplement: Supplementary file 4 — Supplementary file4 (JPEG 131 kb) [file 464_2022_9201_MOESM4_ESM.jpeg]

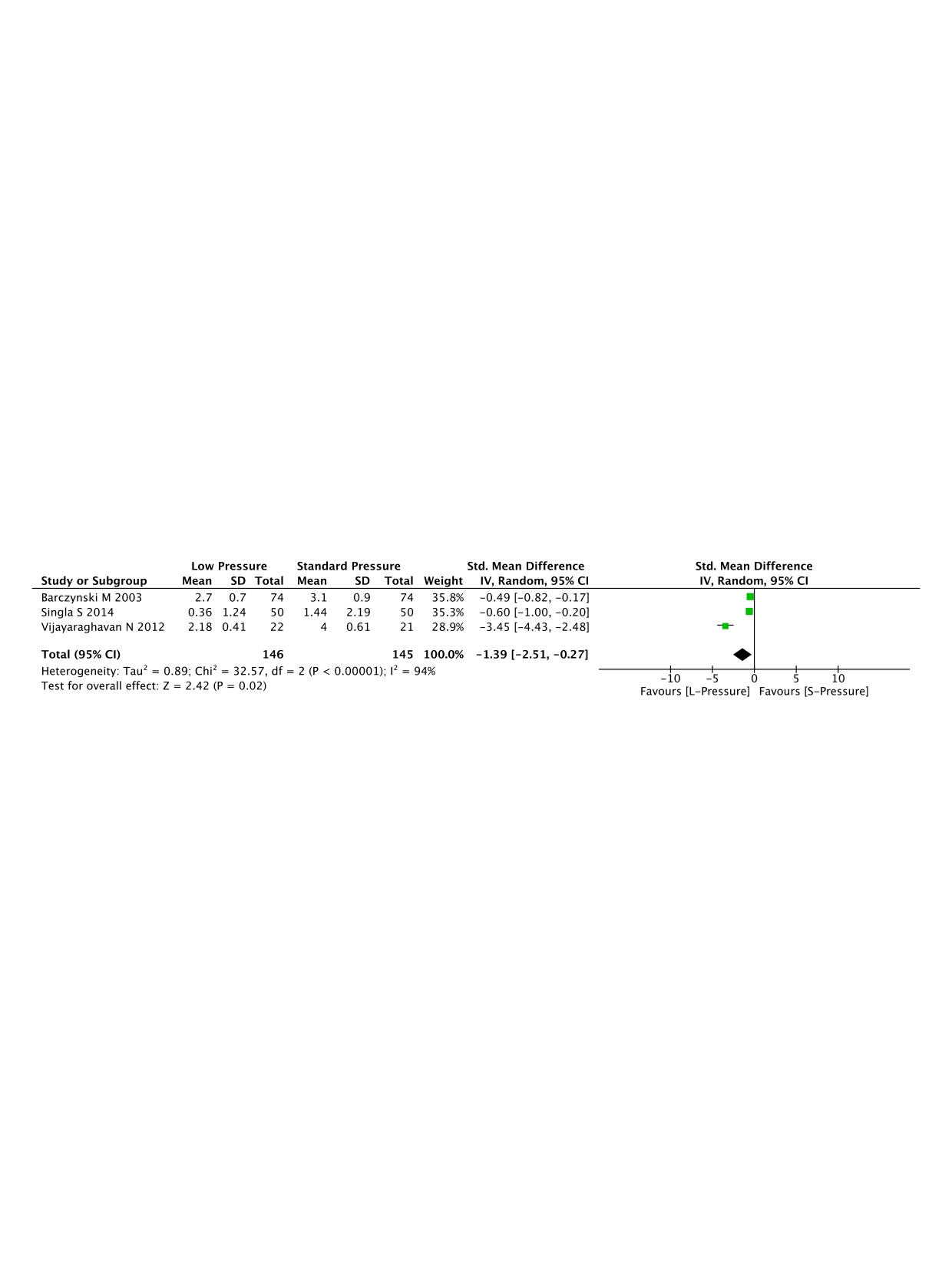

Supplement: Supplementary file 5 — Supplementary file5 (JPEG 141 kb) [file 464_2022_9201_MOESM5_ESM.jpeg]

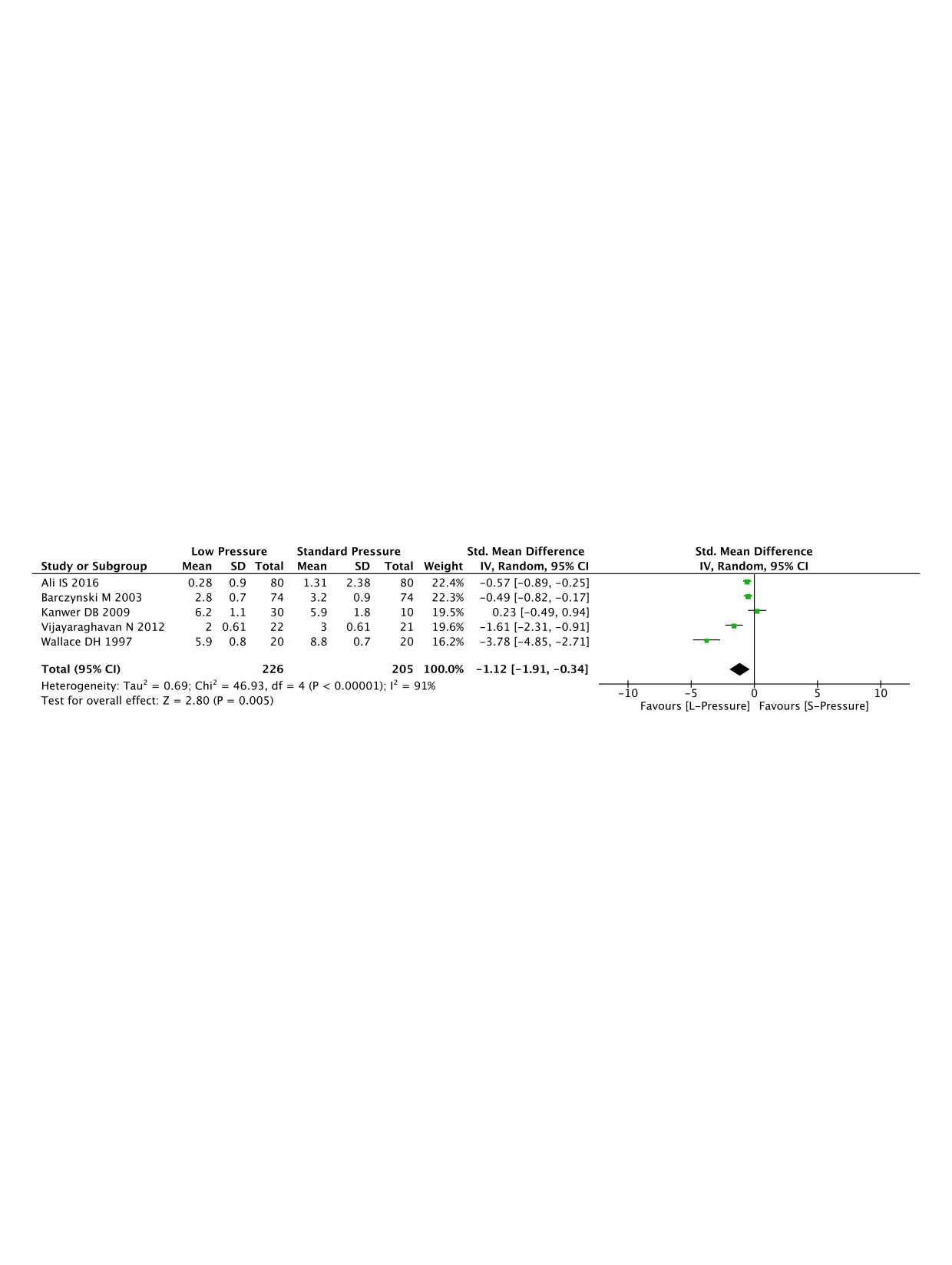

Supplement: Supplementary file 6 — Supplementary file6 (JPEG 161 kb) [file 464_2022_9201_MOESM6_ESM.jpeg]

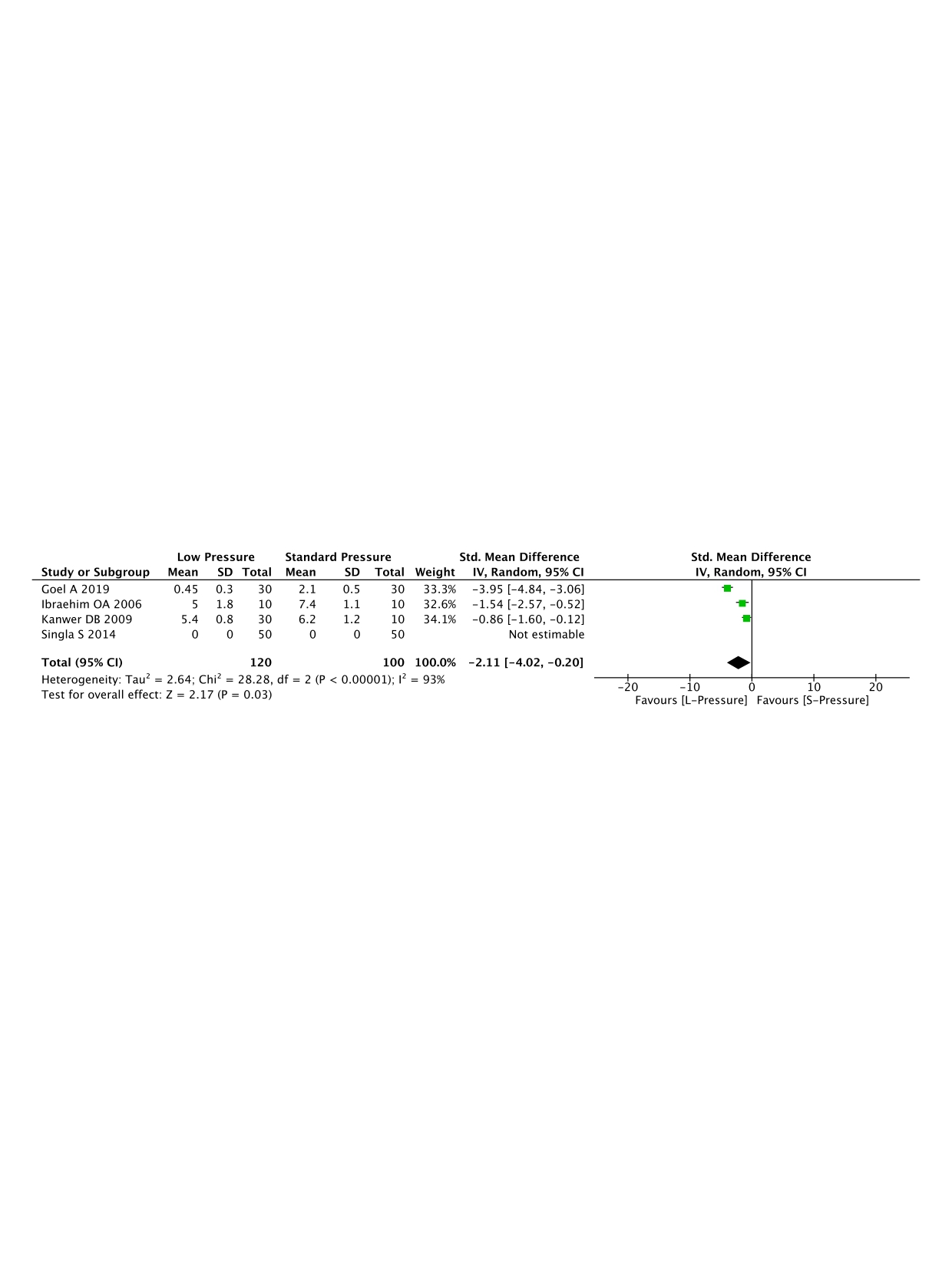

Supplement: Supplementary file 7 — Supplementary file7 (JPEG 148 kb) [file 464_2022_9201_MOESM7_ESM.jpeg]

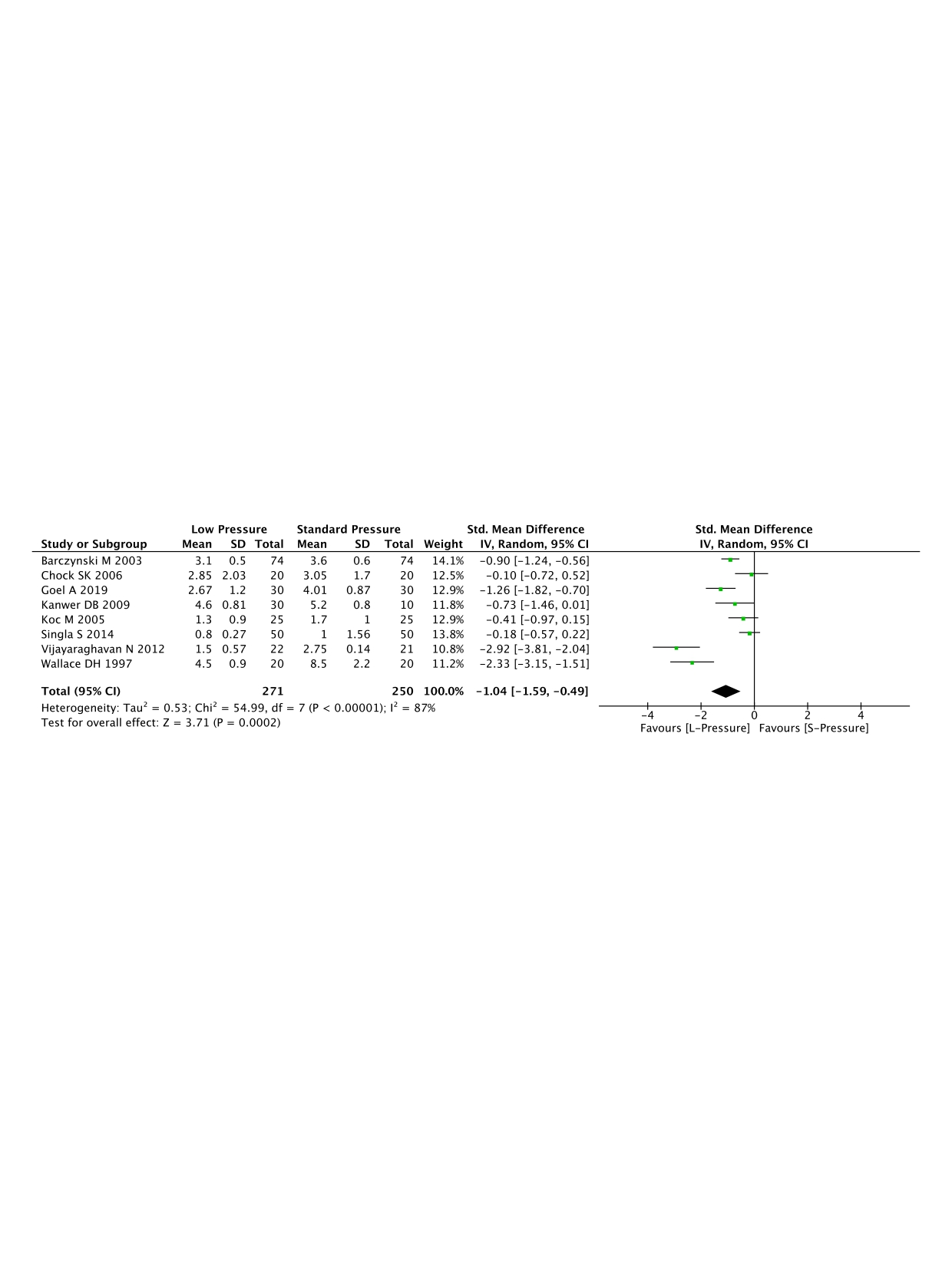

Supplement: Supplementary file 8 — Supplementary file8 (JPEG 196 kb) [file 464_2022_9201_MOESM8_ESM.jpeg]

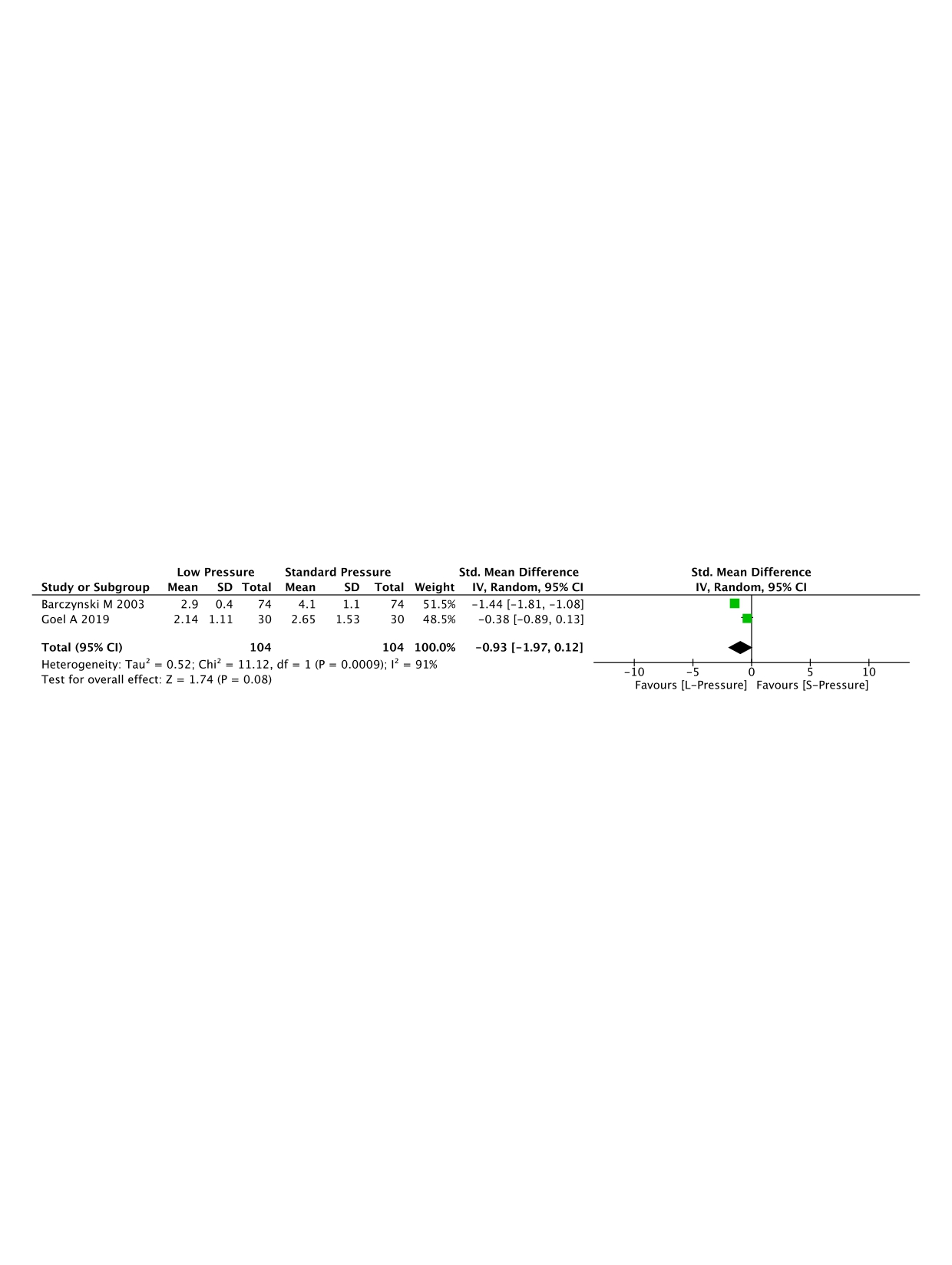

Supplement: Supplementary file 9 — Supplementary file9 (JPEG 130 kb) [file 464_2022_9201_MOESM9_ESM.jpeg]

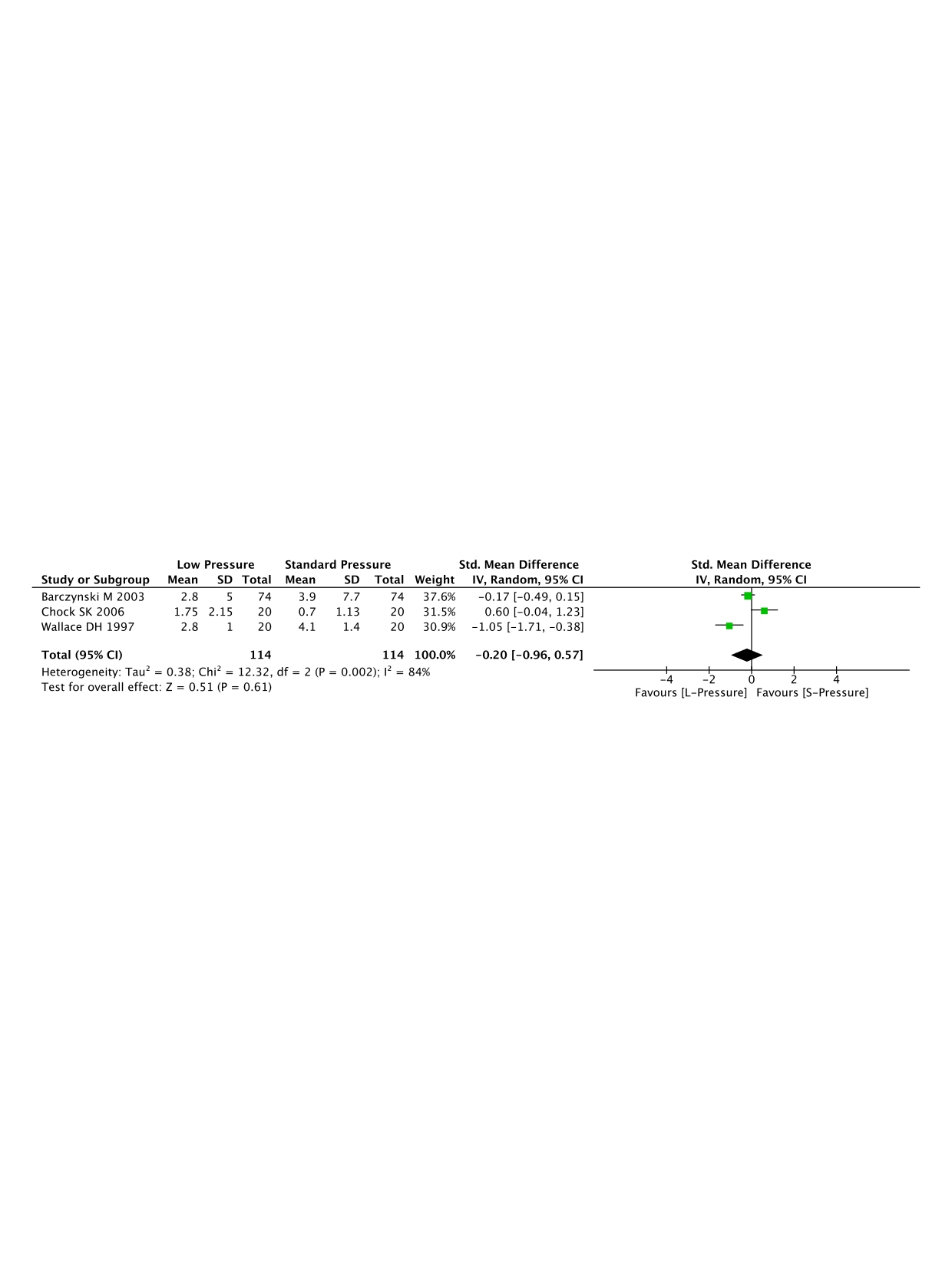

Supplement: Supplementary file 10 — Supplementary file10 (JPEG 141 kb) [file 464_2022_9201_MOESM10_ESM.jpeg]

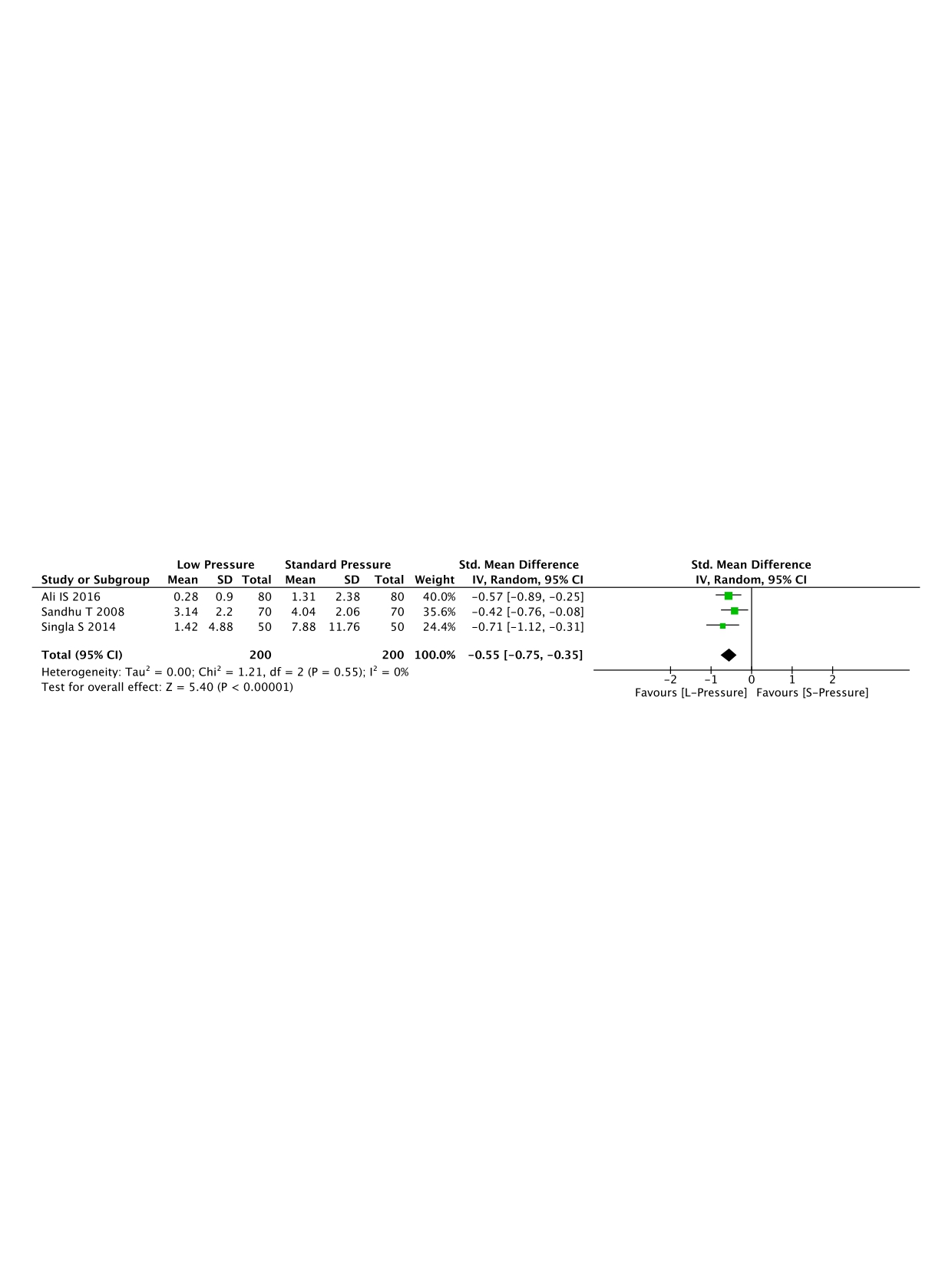

Supplement: Supplementary file 11 — Supplementary file11 (JPEG 142 kb) [file 464_2022_9201_MOESM11_ESM.jpeg]

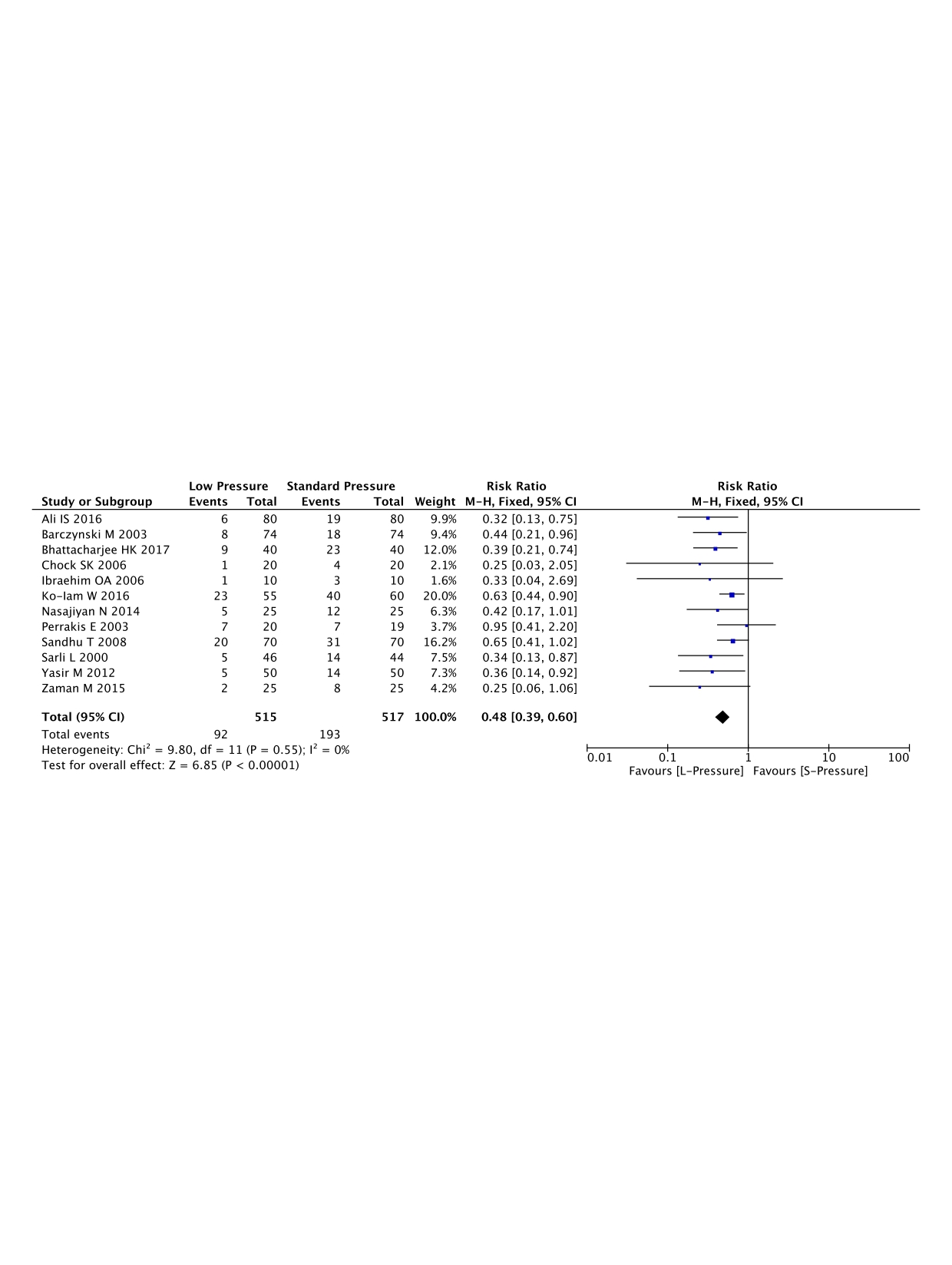

Supplement: Supplementary file 12 — Supplementary file12 (JPEG 225 kb) [file 464_2022_9201_MOESM12_ESM.jpeg]

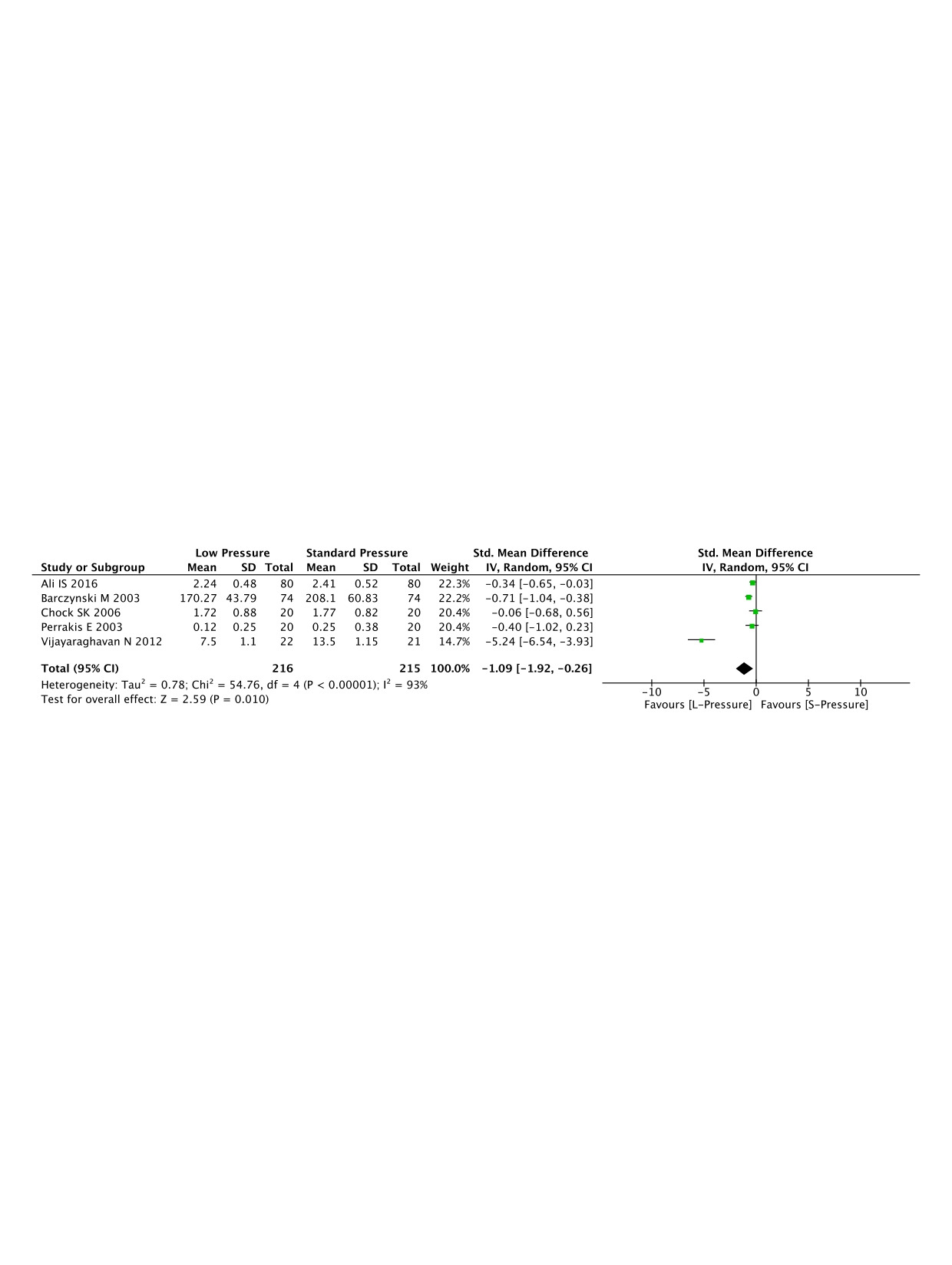

Supplement: Supplementary file 13 — Supplementary file13 (JPEG 163 kb) [file 464_2022_9201_MOESM13_ESM.jpeg]

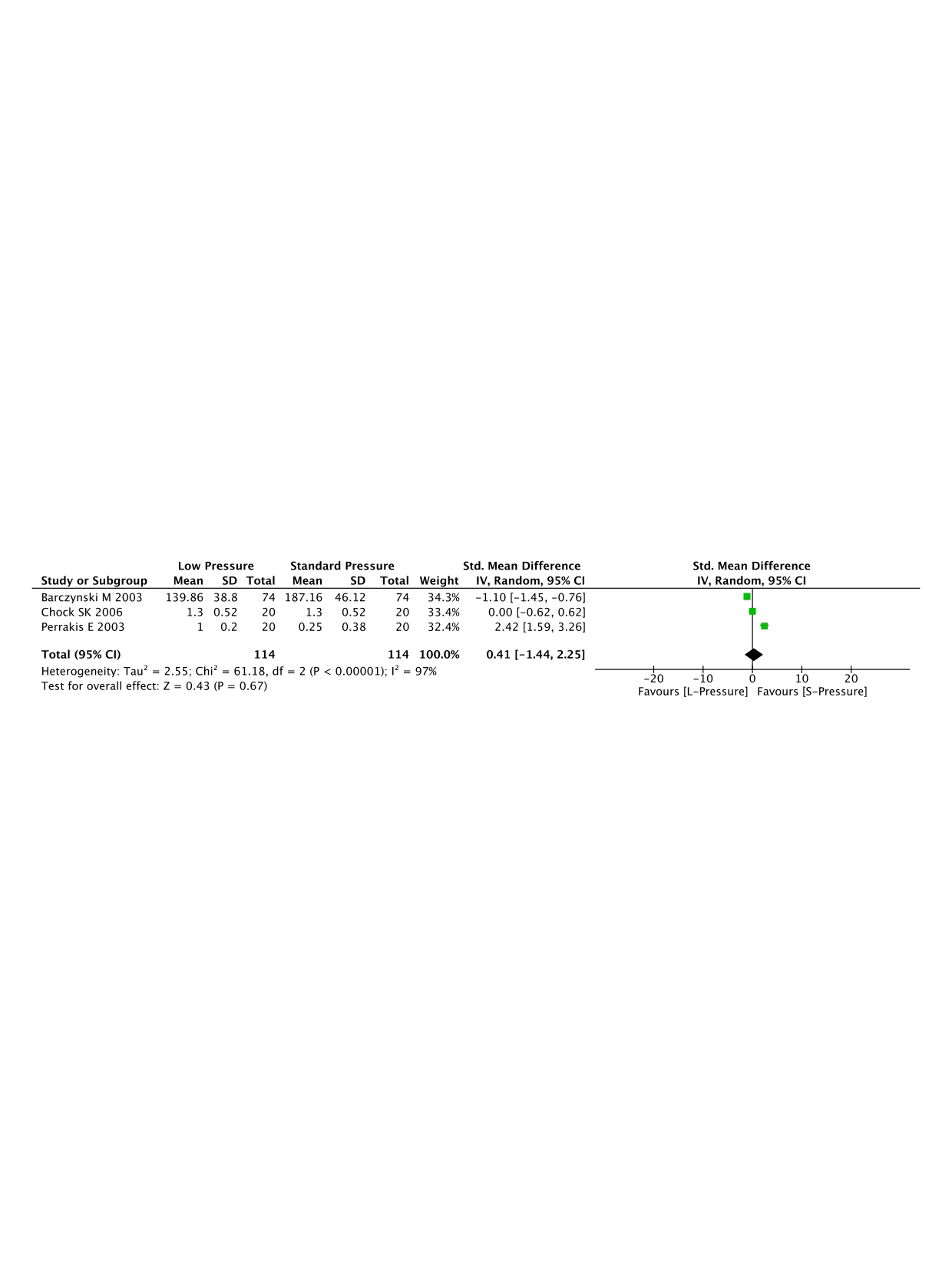

Supplement: Supplementary file 14 — Supplementary file14 (JPEG 142 kb) [file 464_2022_9201_MOESM14_ESM.jpeg]

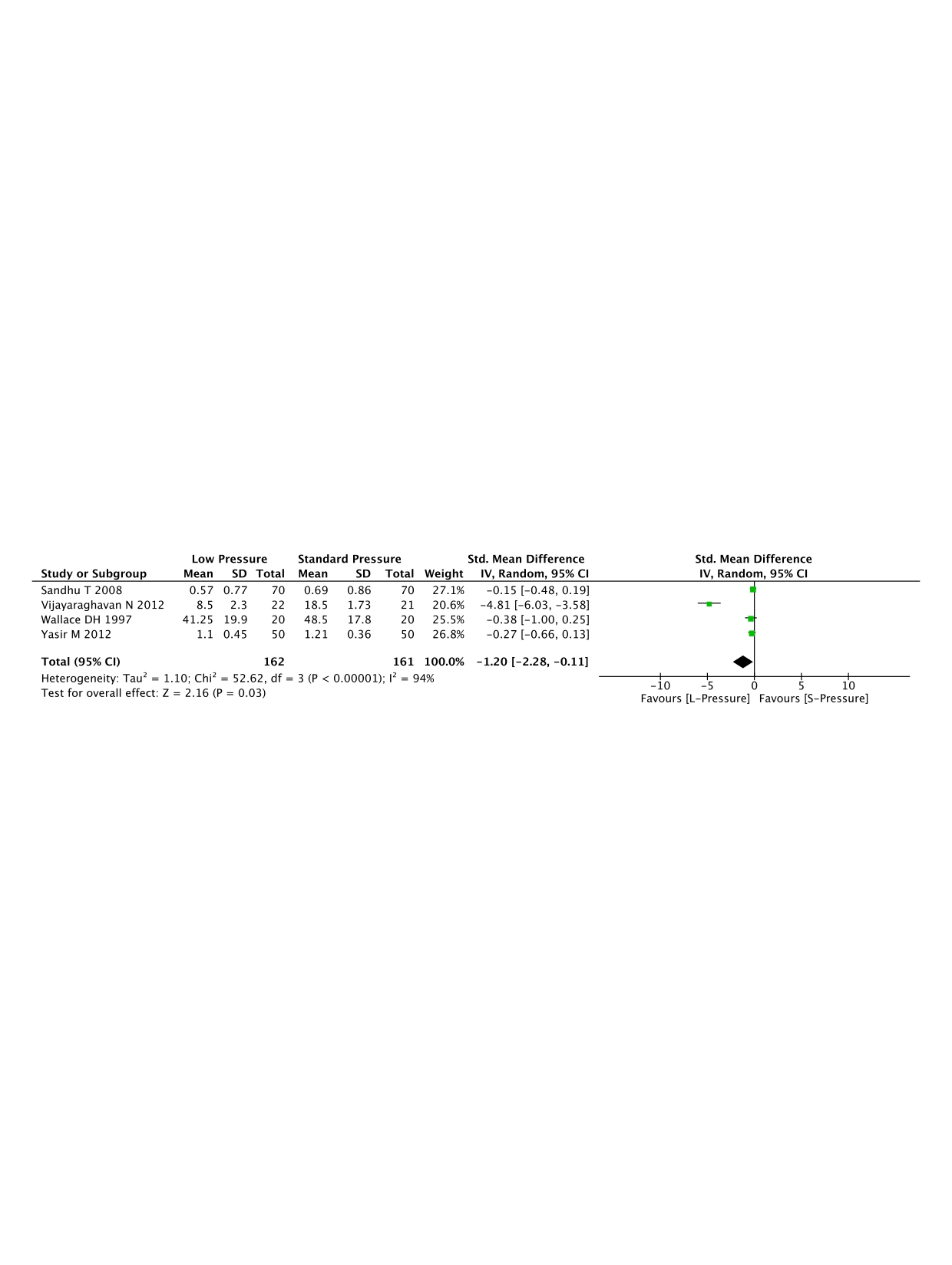

Supplement: Supplementary file 15 — Supplementary file15 (JPEG 153 kb) [file 464_2022_9201_MOESM15_ESM.jpeg]

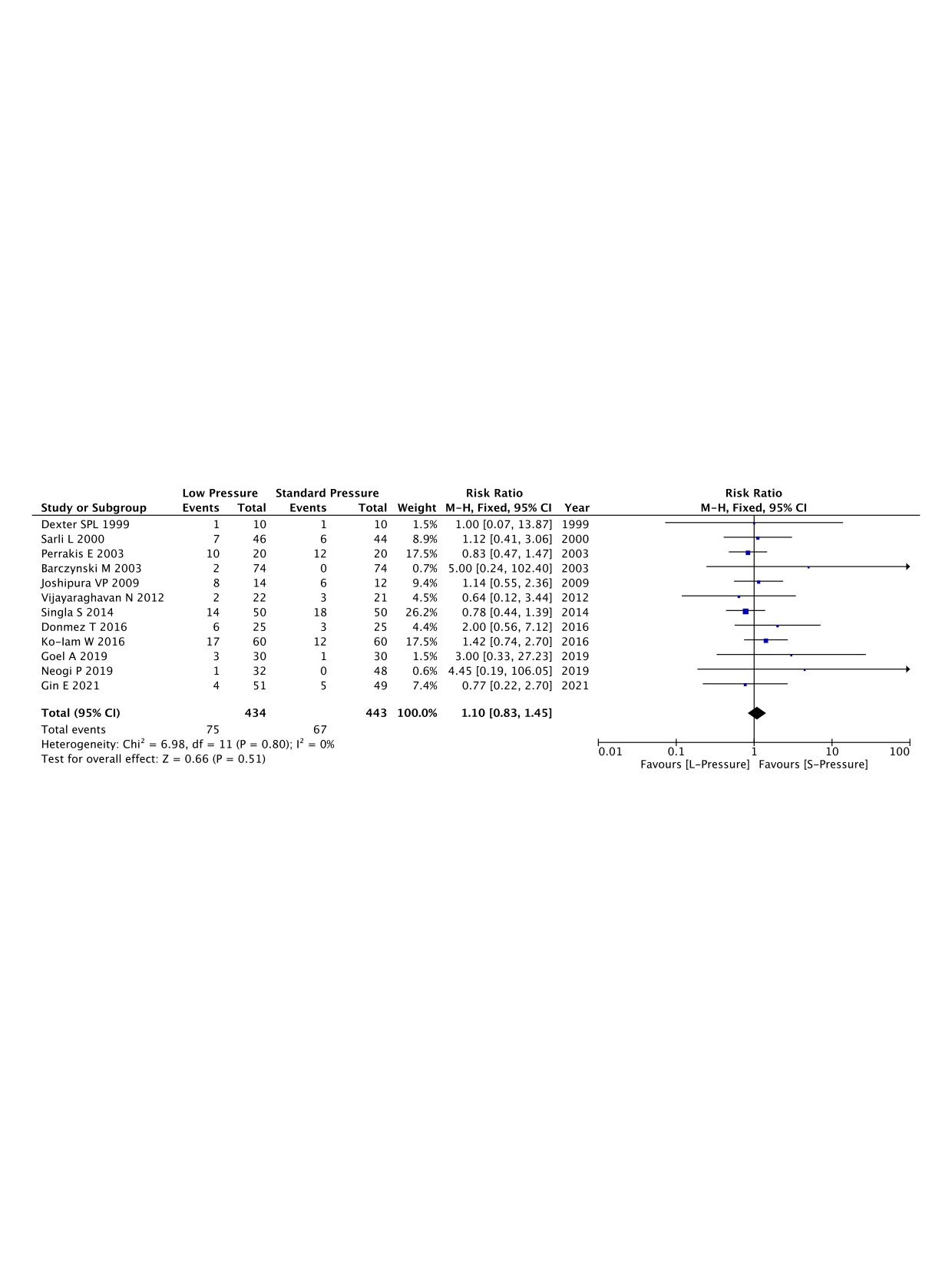

Supplement: Supplementary file 16 — Supplementary file16 (JPEG 220 kb) [file 464_2022_9201_MOESM16_ESM.jpeg]

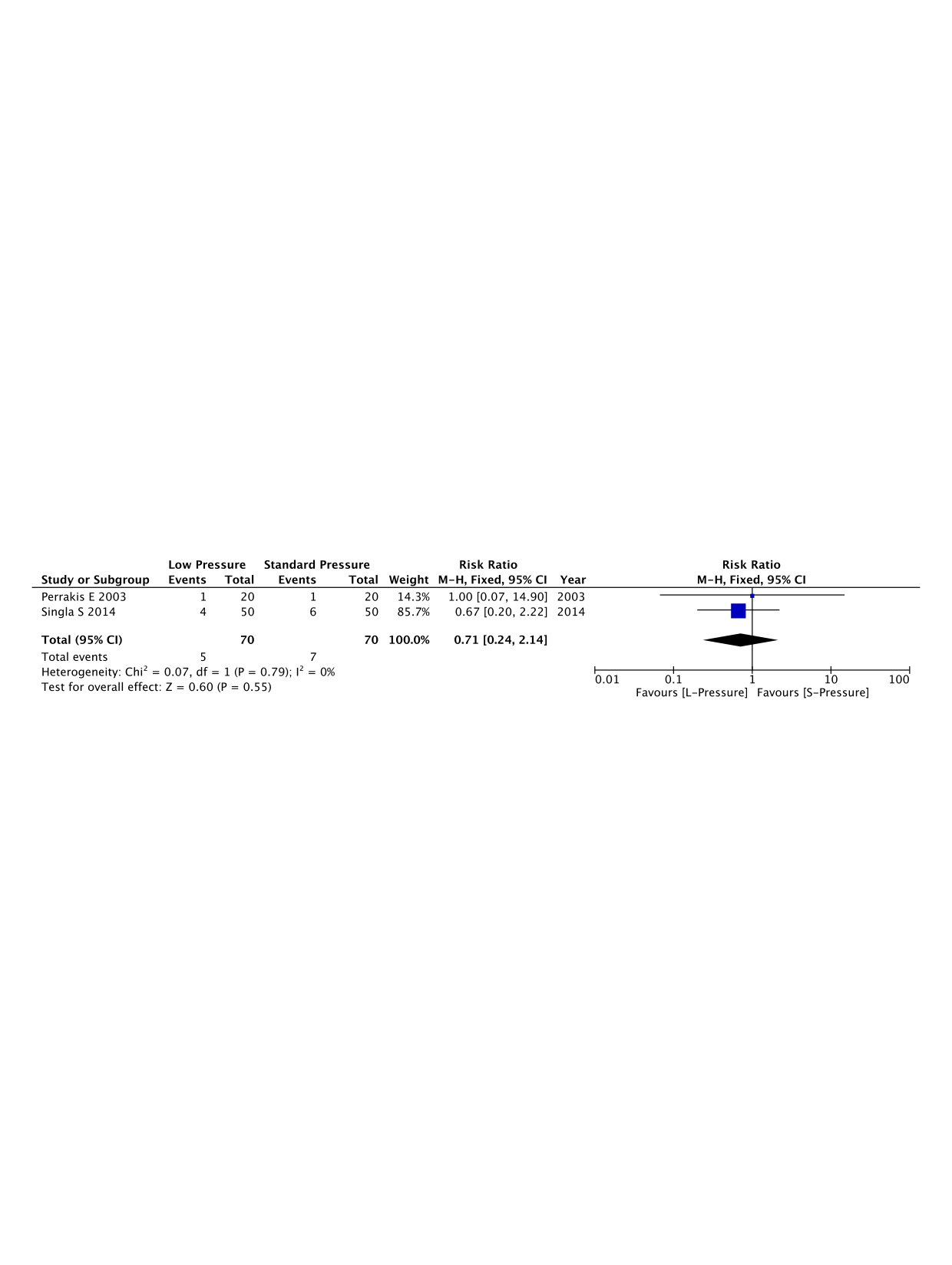

Supplement: Supplementary file 17 — Supplementary file17 (JPEG 125 kb) [file 464_2022_9201_MOESM17_ESM.jpeg]

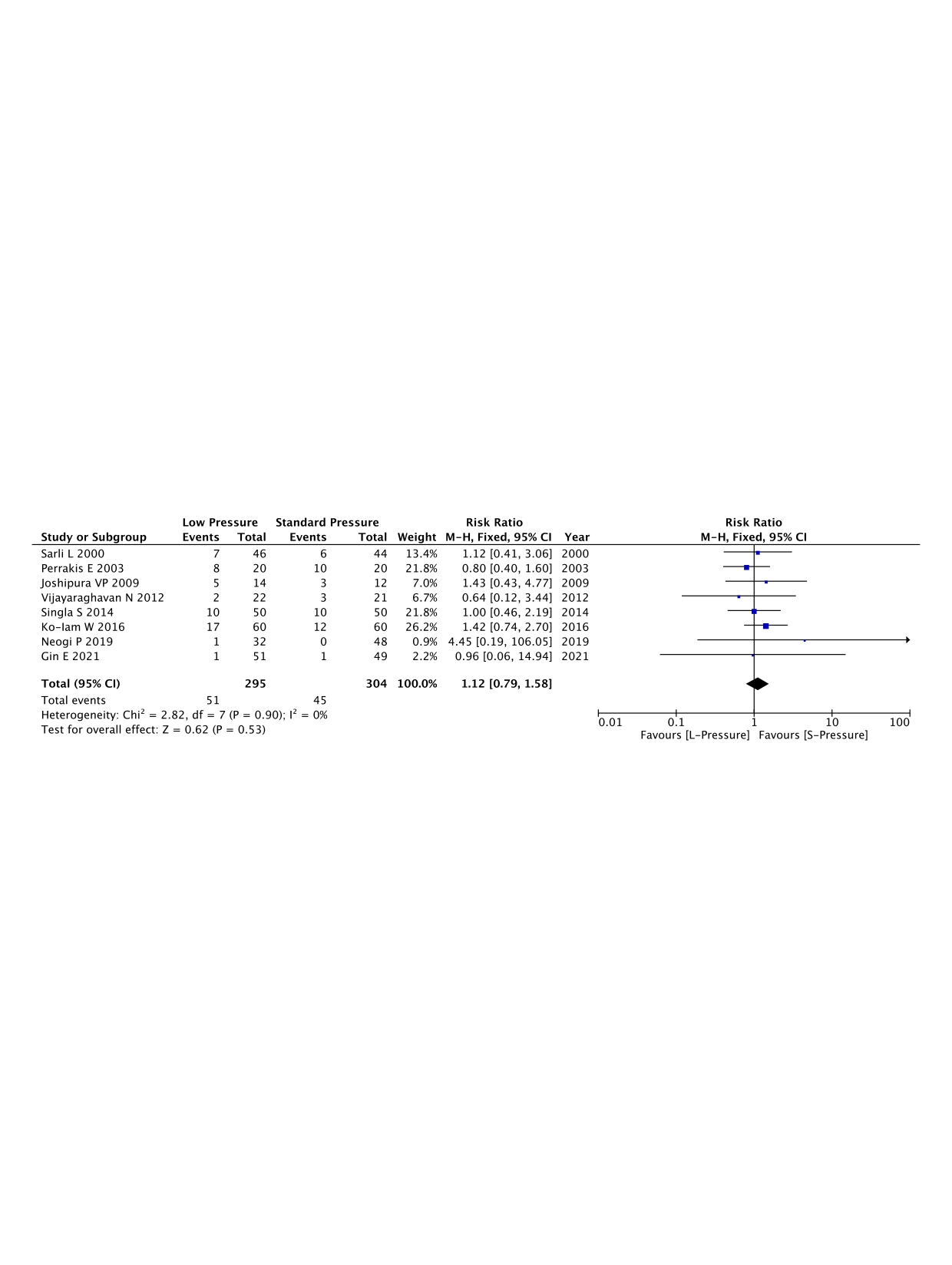

Supplement: Supplementary file 18 — Supplementary file18 (JPEG 181 kb) [file 464_2022_9201_MOESM18_ESM.jpeg]
