# Supplementary material for: Low-pressure versus standard-pressure pneumoperitoneum in laparoscopic cholecystectomy: a systematic review and meta-analysis of randomized controlled trials
Source: Surg Endosc. 2022 Apr 18;36(10):7092–113. doi: 10.1007/s00464-022-09201-1 (PMC9485078; doi:10.1007/s00464-022-09201-1)
Supplement: Supplementary file 19 — Supplementary file19 (DOCX 30 kb) [file 464_2022_9201_MOESM19_ESM.docx]

| **Author (year) [ref]** | **Functional outcomes** |
| --- | --- |
| Ekici Y (2009) [16] | **QTd and QTcd:** increase with a statistical difference during CO2 insufflation in both groups (which are associated with an increased risk of arrhythmias  and cardiac events). They were significantly higher in the HPG than in LPG.  **HR:** temporary increase with statistical difference in HGP, who decrease after desufflation |
| Ibraehim O.A. (2006) [17] | **MAP and HR, ETCO2, SaO2, pH**: NS in two groups  **Bicarbonate/lactate level at 30’ insufflation**: 24.55 ± 5.8 vs 55.7 ± 8.6 HPG, p=0.04/ 1.05 ± 0.251 vs 1.69 ± 0.545 in HPG p=0.03  **Bicarbonate/lactate level at end of surgery**: 23.02 ± 1.29 vs 21.6 ± 1.068 in HPG / 1.25 ± 0.481 vs 2.77 ± 0.641 in HPG  **Lactate level at 1 hr postop**: 1.31 ± 0.398 vs 2.907 ± 0.862 in HPG, p<0.001 |
| Joshipura V.P. (2009) [18] | **i.op pO2 level at 30min inflation**: 206.27 ± 17.07 in HPG and 181.48 ± 17.78 in LPG, p<0.001;  **i.op pO2 level after deflation of pneumoperitoneum**: 196.31 ± 13.62in HPG and 231.69 ± 21.52in LPG, p<0.001.  **arterial bicarbonate levels and base excess levels**: NS in two group analgesic consumption  **pulmonary function (FEV1, FVC, MVV)**  **cardiac function** (2D echocardiography, ECD lower limb venous systems): NS in two groups  **Surgeon difficulties**: NS in two groups |
| Koc M. (2005) [19] | **Total gas volume**: NS in two groups  **Time to return to oral food**: NS in two groups |
| Perrakis E. (2003) [20] | **Tissue trauma (CRP and WBC)**: NS between two groups  **Operating Difficulties**: NS between two groups |
| Wallace DH. (1997) [21] | **Cardiac function** (Intraop heart rate or cardiac index): NS differences, nut greater and lasted longer in High pressure group.  **MAP**: rose significantly in both group.  **pulmonary function** ( FEV1, FVC, peak expiratory flow rate): NS difference |
| Barczynski M. (2002) [27] | **MAP**: during insufflation 82.9±9.8 LPG vs 97.8±12.8 HPG, p<0.05;  **HR**: NS in two groups  **Sympathetic activation**: low frequency 68.12±6.56 LPG vs 78.26±5.43 HPG, p<0.01, high frequency 15.56±2.54 LPG vs 24±3.48 HPG, p<0.05, |
| Barrio J. (2017) [49] | **Good vs bad surgical condition**: *G1vsG2: p=NS in surgical time; G2vs G3: p=NS in surgical time  **Dissection of the gallbladder time-point**: G1vsG2: p=NS; G2vs G3: 19 n of pts (63.3%) vs 29(96.7%) p=0.002 **Exposure of the gallbladder time-point**: G1vsG2: p=NS; G2vs G3: 24 (80%) vs 30(100%) p=0.024 **Extraction, hemostasis, closure time-point**: G1vsG2: p=NS; G2vs G3: 20 (76.9%) vs 29(96.7%) p=0.04 |
| Bhattacharjee HK (2017)  [29] | **HR, SBP, DBP**: NS in two groups |
| Celik AS. (2010) [56] | **anesthesia duration**: 42.1±8.4 LPG vs 49.6±8.5 HPG, p=0.006; 40.3±5.1 SPG vs 49.6±8.5 HPG, p=0.0004; 42.1±8.4 LPG vs 40.3±5.1 SPG, p=NS in two  groups (statistically significant difference was found between the groups low- pressure and HP and SP and HP, and statistically significant difference was found regarding operative duration between the groups SP and HP. There was no difference between the other groups). |
| Karagulle E. (2009) [22] | **Blood pH**: NS in two groups  **pCO2 levels**: NS in two groups  **Bicarbonate levels**: NS in two groups  **Lactate levels**: NS in two groups |
| Kanwer et al (2009) [31] | **SBP, DBP**: NS in two groups |

|  | **HR**: NS in two groups |
| --- | --- |
| Morino et al (1998) [32] | **AST, ALT, total bilirubin, prothrombin** at 6-24-48-72h: significant higher in LPG (increase of AST and ALT was statistically significant and correlated both to the level (10 versus 14 mmHg) and the duration of pneumoperitoneum.)  LPG with pnp lasted 30 min at 6h: AST 43.57 ± 14.43 UI/l and the ALT 32 ± 12.52 UI/l. LPG with pnp lasted 90 min at 6h: AST 63.64 ± 20.21 UI/l and ALT was 51.43 ± 22.17 UI/l . **HPG**: AST 170.33 ± 28.34 UI/l and the ALT 113.33 ± 20.41 UI/l. |
| Hasukič Š. (2005) [23] | **ALT** after 24 h (Low: 1473.72 ± 654.85; High: 2233.74 ± 1247.33; p = 0.0096) and 48 h (Low: 1322.99 ± 601.51; High 2007.80 ± 747.55; p = 0.0008)  **AST** after 24 h (Low: 1189.96 ± 404.79.; High: 1679.40 ± 766.13; p = 0.0069) were increased in the patients who underwent High pressure.  **Total bilirubin, ALP, and GGT levels**: NS in two groups |
| Donmez T. (2016) [24] | **PT, TT, aPTT, INR, and D-dimer and fibrinogen levels** significantly increased after the surgery in both of the groups. D-dimer level was significantly higher in 14-mmHg group at post24.  D-Dimer Low Post1 0.56 ± 0.22 High0.68 ± 0.24 <0.001  D-Dimer Low Post24 0.91 ± 0.34 High 1.51 ± 0.30 <0.001 |
| Filho MAM (2021) [25] | **Neutrophil gelatinase-associated lipocalin (NGAL) and cystatin C levels** measured at the beginning of the procedure (T0), at the end of the procedure (T1), and 24 hours after the procedure (T2): significant difference between the time points with elevation at T1 (P < 0.001) NGAL.  **Cystatin C** had an elevation at T1 in both groups (P = 0.021)  **NGAL** (ng/dL) T0 60.4 ± 11.3 T1 153.6 ± 19.3 T2 62.3 ± 10.4  **Cystatin C** (ng/dL) T0 2324.3 ± 540.8 T1 2677.7 ± 927.4 T2 2384.2 ± 500.6 |
| Dexter SPL (1999) [26] | **HR and MBP**: increased in HPG during insufflation.  **Stroke volume (SV) and cardiac output:** were depressed by a maximum of 26% and 28% (SV 0.1 > p > 0.05, cardiac output p > 0.1).  In the low-pressure group, insufflation produced a rise in MBP and a peak rise in both stroke volume and cardiac output of 10% and 28%, respectively (p < 0.05). |
| Gupta R. (2013) [36] | **Bilirubin, AST, and ALT levels**: significantly higher on POD 1 in the Standard group (P = 0.0179, 0.0001, and 0.0001, respectively) but normalized on POD 7. |
| Goel A. (2019) [37] | **HR before and during insufflation**: NS in two groups, after 15 minutes of insufflation of CO2 and till exsufflation heart rate was significantly lower in LPG.  **Mean systolic and diastolic pressure**: NS in two groups before and during insufflation, but pressures stayed significantly low during surgery until exsufflation in LPG.  **End-tidal CO2**: significantly lower in LPG p after insufflation. |
| Mohammadzade AR (2016) [39] | **MSBP and HR**: higher in HPG than LPG, p<0.05;  **diastolic pressure:** p=NS  **mean of ALP:** higher in LPG than HPG, p<0.05  **ALT, AST, total and direct bilirubin:** p=NS |
| Nasajiyan N. (2014) [40] | **Antiemetic drug consumption**: NS |
| Shoar S. (2015) [42] | **Serum levels of cortisol (microg/L), Glucose level (mg/dl), CRP (mg/l), Adrenaline (pg/l):** not differ significantly between HPG and LPG  **HR and MAP:** slightly higher in HPG (p<0.05 only for HR at 30 min intraoperatory)  **Intravenous volume:** 1928±464.15 cc vs 1892±593.6 cc, p=NS  **Urine:** 272±153.48 cc vs 296±202 cc.1, p=NS |

|  | The serial changes of serum levels of cortisol, glucose, adrenaline, and CRP were compared between surgery day post op 6-hr and 1^st^ postoperative day, which did not differ significantly between the standard-pressure and the low-pressure groups. However, when the within group changes of the serial values of heart rate, mean arterial pressure, end-tidal CO2, serum glucose, CRP, cortisol, and adrenaline were evaluated, there were a significant increase in the end-tidal CO2, serum glucose, and CRP after surgery in both low- pressure and standard pressure groups (p<0.05). Changes in heart rate, mean arterial  pressure, cortisol and adrenaline were not significant during the time period in both groups (p>0.05) |
| --- | --- |
| Torres K. (2009) [43] | **serum concentrations of IL-6:** increased significantly after the operations in both groups at 6 hr and 24 hr. Increase at 48 hr in LPG.  **serum concentrations of IL-8, IL-10, IL-6:** No differences before surgery between the groups.  **VEGF-A:** at 6 and 48 h were significantly lower in LPG.  **Endostatin:** No significant variations were observed in endostatin serum concentration. |
| Umar A. (2013) [54] | **Mean HR:** increased immediately during insufflation, 5, 10, 20, and 30 min after insufflation and decreased at exsufflation and 10 min after exsufflation in all the three groups; was statistically significant at 10 and 20 min after CO2 insufflation, and highly significant at 30 min after CO2 insufflation, at exsuf- flation, and 10 min after exsufflation.  **SBP:** increased immediately during insufflation, 5, 10, 20, and 30 min after insufflation and decreased at exsufflation and 10 min after exsufflation in all the three groups. The difference was statistically significant, immediately during insufflation and highly significant at 5, 10, 20 and 30 min after CO2 insufflation, at exsufflation, and 10 min after exsufflation.  **MAP:** increased during insufflation, 5, 10, 20, and 30 min after insufflation and decreased at exsufflation and 10 min after exsufflation in all the three groups. The difference was statistically significant immedi- ately during insufflation and highly significant at 5, 10, 20, and 30 min after CO2 insufflation, at exsufflation, and 10 min after exsufflation.  **EtCO2:** increased immediately after insuffla- tion and the rise in EtCO2 continued with the increasing period of CO2 insufflation and even at 10 min after exsuf- flation the mean values were higher than the base line in all the three groups. The difference was statistically highly significant at 5, 10, 20, and 30 min after CO2 insufflation at exsufflation and 10 min after exsufflation.  **HP due to CO2 insufflation is associated with more fluctuations in hemodynamic parameters and increased peritoneal absorption of CO2 as compared to LP.** |
| Vijayaraghavan N. (2012)  [45] | **The liver function ( AST, ALT, ALP, Bilirubine)**: the only significative changes were in AST and ALT in HPG (p=0.04 and p=0.01 respectively).  **CRP levels:** varied significantly only at 24 hours postoperatively (P = 0.001) |
| Basgul E. (2004) [33] | **Serum IL-2:** showed a significant decrease before the incision in HPG and a significant increase after surgery and 24 hours postoperatively to reach pre- induction levels in the LPG (p<0.001)  **Serum IL-6:** increase at the end of surgery and postoperatively was lower in LPG (p<0.001) |
| Topal A. (2011) [55] | **TEG (maximum amplitude (MA), a-angle, reaction time (R), and K time (K).) 1 hour before the induction of anesthesia, the 30th minute intraoperatively, and the 24th hour postoperatively.**  **Hemoglobin, hematocrit (Htc), thrombocyte count, PT, aPTT, INR 1 hour before the induction of anesthesia, the 30th minute intraoperatively, and the 24th hour postoperatively.**  In the postoperative 24th hour, the R-value was significantly lower in group 3 than that in group 1 (10 mmHg) and group 2 (13 mmHg) (P<0.05). Increased values of MA intraoperative 30th minute and postoperative 24th hour in group 3 (16 mmHg)) with respect to group 1 (P<0.05) and postoperative 24th hour, and the MA value in group 3 was significantly higher than those of group 2 (P<0.05). In group 3, the a-angle was significantly higher than that of group 1  and group 2 at intraoperative 30th minute and postoperative 24th hour (P<0.05). In postoperative 24th hour, the K-value was significantly lower in group 3 than in group 2 and group 1 (P<0.05). |

| Neogi P. (2019) [4] | **Mean bilirubin:** pre-operatively is comparable in both groups. Decrease in mean serum bilirubin level in both groups in immediate postoperative period (P  < 0.05). Increase in its level was observed after 24 hours in the SPG group, but not in the LPG group (P<0.05). Day 7 bilirubin level was normal in both the groups.  **Mean SGOT, SGPT, LDH and ALP:** SGOT and LDH increased significantly (P < 0.05) in immediate postoperative period and further increase after 24 hours (P < 0.05). They returned to normal value on the seventh day. No significant change in levels of ALP in either group. |
| --- | --- |
| Polat C. (2003) [35] | Serum protein carbonyls and TBARS levels were found to be increased immediately after desufflation in both study groups when compared to the pre- operative levels. On the other hand, protein sulfhydryl levels were found to be decreased in both study groups. Although increases in protein carbonyls and TBARS levels were more prominent in patients in HPG, this difference was not statistically significant between both groups. Both intraabdominal pressure,  15 and 10 mmHg, could lead to an increased oxidative stress response, but no difference between two groups. |
| Sefr R. (2003) [46] | **Blood pH:** increased slightly with pneumoperitoneum and decreased after 30 min, reaching its lowest level in the recovery room. Then, a gradual increase toward normal values was noticed. No significant differences were found between the two groups  **PaO2 levels:** in arterial blood increased at the beginning and after decrease, p=NS  **PaCO2 levels:** did not increase with pneumoperitoneum.  **Bicarbonate:** decreased moderately during the operation in both groups, p=NS  **Base excess:** decreased in the LPG and increased in the HPG during the first 60 min, p=NS |
| Eryılmaz HB (2012) [8] | **ICG-PDR 2 values:** were found to be 25.63 ± 2.1% per min in LPG versus 19.06 ± 2.2% per min in HPG (P < 0.05). There was a statistically significant decrease between baseline and postoperative ICG-PDR values in HPG compared to LPG (P < 0.05).  **AST and ALT level:** increase between baseline and postoperative 1st-hour serum in HPG (P < 0.05) compared to LPG, p<0.05. On postoperative 24st- hour serum P=NS  **All the time bilirubin: P=NS** |
| Celik V. (2004) [56] | **gastric intramucosal pH** using gastric tonometry during pneumoperitoneum 30 minutes following insufflation and 1 hour after the ending of the surgery:  p=NS in 5 groups (8-10-12-14-16 mmHg). |

**TABLE 5: Functional outcomes reported in 33 RCTs included in the systematic review.**

Legend: Pts: patients; IAP: intra-abdominal pressure; N: number; yrs: years; post-op: postoperative; min: minutes; NS: not significative; HPG: high pressure group; LPG: low pressure group; CRP: c-reactive protein; WBC: white blood cell count; ^: p<0-05; MAP: mean arterial pressure; FVC: forced vital capacity; FEV1: Forced Expiratory Volume in 1s; NS: not significat; MVV: minute ventilatory volume; ECD: echocolordoppler; LPG: low pressure group; HPG: high pressure group; SPG: standard pressure group; QTc: rate-correct QT interval; QTd: QT dispersion; QTcd: corrected QT dispersion; SBP: systolic blood pressure; DBP: diastolic blood pressure; HR: heart rate; MBP: mean blood pressure; MSBP: mean systolic blood pressure; NR: not reported; IL: interleukine; VEGF: vascular-endothelial growth factor; ***:** Group 1(G1) LP + deep- NMB (1-5 PTC), Group 2 (G2) LP + moderate- NMB (1–3 TOF), Group 3 (G3): standard pressure; ALP: alkaline phosphatase; ICG-PDR 2: indocyanine green; TEG: thromboelastograph; EtCO2: End-tidal carbon dioxide; SGOT: serum glutamicoxaloacetic transaminase; SGPT: serum glutamic-pyruvic transaminase; LDH: lactate dehydrogenase; TBARS: thiobarbituric acid reactive substances; HP: high pressure; LP: low pressure.

Thirty-three out of 44 included studies reported functional outcomes (Ekici 2009; Ibraehim 2006; Joshipura 2009; Koc 2005; Perrakis 2003; Wallace 1997; Barczynski 2002; Barrio 2017; Bhattacharjee 2017; Celik 2010; Karagulle 2009; Kanwer 2009; Morino 1998; Hasukič 2005; Donmez 2016; Filho 2021; Dexter 1999; Gupta 2013; Goel 2019; Mohammadzade 2016; Nasajivan 2014; Shoar 2015; Torres 2009; Umar 2013; Vijayaraghavan 2012; Basgul 2004; Topal 2011; Neogi 2019; Polat 2003; Sefr 2003; Eryılmaz 2012; Celik 2004) (**Table 5**). Electrocardiogram, laboratory assays, pulmonary and cardiac function, gas volume, tissue trauma, arterial pressure were evaluated to obtain functional outcomes (**Table 5**).
