# Supplementary material for: Low-pressure versus standard-pressure pneumoperitoneum in laparoscopic cholecystectomy: a systematic review and meta-analysis of randomized controlled trials
Source: Surg Endosc. 2022 Apr 18;36(10):7092–113. doi: 10.1007/s00464-022-09201-1 (PMC9485078; doi:10.1007/s00464-022-09201-1)
Supplement: Supplementary file 20 — Supplementary file20 (DOC 86 kb) [file 464_2022_9201_MOESM20_ESM.doc]

| **Reference** | **Overall risk of bias** | **Domain I**  ROB arising from the randomization process | **Domain II**  ROB due to deviations from the intended interventions (effect of assignment to intervention) | **Domain II**  ROB due to deviations from the intended interventions (effect of adhering to intervention) | **Domain III**  Missing outcome data | **Domain IV**  ROB in measurement of the outcome | **Domain V**  ROB in selection of the reported result |
| --- | --- | --- | --- | --- | --- | --- | --- |
| 15 | Low | Low | Low | Low | Low | Low | Low |
| 16 | Low | Low | Low | Low | Low | Low | Low |
| 17 | Low | Low | Low | Low | Low | Low | Low |
| 18 | Low | Low | Low | Low | Low | Low | Low |
| 1 | Low | Low | Low | Low | Low | Low | Low |
| 27 | Low | Low | Low | Low | Low | Low | Low |
| 19 | Low | Low | Low | Low | Low | Low | Low |
| 20 | Low | Low | Low | Low | Low | Low | Low |
| 21 | Low | Low | Low | Low | Low | Low | Low |
| 28 | Low | Low | Low | Low | Low | Low | Low |
| 29 | Low | Low | Low | Low | Low | Low | Low |
| 23 | Low | low | low | low | low | Low | Low |
| 31 | Low | low | Some concerns | Some concerns | Low | some concerns | low |
| 30 | High | Some concerns | High | High | Low | some concerns | Low |
| 32 | Low | Some concerns | some concerns | some concerns | Low | low | Low |
| 22 | Low | Low | Low | Low | Low | Low | Low |
| 26 | Low | Low | Low | Low | Low | Low | Low |
| 24 | Low | Low | Low | Low | Low | Low | Low |
| 25 | Low | Low | Low | Low | Low | Low | Low |
| 53 | Low | Low | Low | Low | Low | Low | Low |
| 36 | Low | Low | Low | Low | Low | Low | Low |
| 38 | Low | Low | Low | Low | Low | Low | Low |
| 36 | High | High | High | High | Low | some concerns | some concerns |
| 40 | some concerns | Some concerns | Low | Low | Low | some concerns | some concerns |
| 42 | Low | Low | Low | Low | Low | Low | Low |
| 43 | some concerns | High | Some concerns | Some concerns | Low | Low | some concerns |
| 45 | Low | Low | Low | Low | Low | Low | Low |
| 44 | some concerns | High | Some concerns | Some concerns | Low | Low | some concerns |
| 52 | Low | Low | Low | Low | Low | Low | Low |
| 49 | Low | Low | Low | Low | Low | Low | Low |
| 48 | Low | Low | Low | Low | Low | Low | Low |
| 47 | Low | Low | Low | Low | Low | Low | Low |
| 4 | Low | Low | Low | Low | Low | Low | Low |
| 54 | Low | Low | Low | Low | Low | Low | Low |
| 38 | Low | Low | Low | Low | Low | Low | Low |
| 50 | Low | Low | Low | Low | Low | Low | Low |
| 36 | Low | Low | Low | Low | Low | Low | Low |
| 51 | Low | some concerns | Some concerns | Some concerns | Low | Low | Low |
| 41 | Low | Low | Low | Low | Low | Low | Low |
| 37 | Low | some concerns | Some concerns | Some concerns | Low | Low | Low |
| 35 | Low | some concerns | Some concerns | Some concerns | Low | Low | Low |
| 46 | Low | Low | Low | Low | Low | Low | Low |
| 34 | Low | Low | Low | Low | Low | Low | Low |
| 55 | Low | some concerns | Some concerns | Some concerns | Low | Low | Low |
| 56 | Low | some concerns | Some concerns | Some concerns | Low | Low | Low |
| 33 | Low | some concerns | Some concerns | Some concerns | Low | Low | Low |
